# Supplementary material for: First Gallium and Indium Crystal Structures of Curcuminoid Homoleptic Complexes: All-Different Ligand Stereochemistry and Cytotoxic Potential
Source: Int J Mol Sci. 2023 Nov 15;24(22):16324. doi: 10.3390/ijms242216324 (PMC10671313; doi:10.3390/ijms242216324)
Supplement: Supplementary file 1 [file ijms-24-16324-s001.zip › ijms-2683867-supplementary.pdf]

The First Gallium and Indium Crystal Structures of Curcuminoid Homoleptic Complexes: All-Different Ligand Stereochemistry and Cytotoxic Potential

William Meza-Morales, Yair Alvarez-Ricardo, Leidys L. Pérez-González, Rosario Tavera-Hernández, María Teresa Ramírez-Apan, Rubén A. Toscano, Rubén Sanchez-Obregón, Marco A. Obregón-Mendoza * and Raúl G. Enríquez *

Instituto de Química, Universidad Nacional Autónoma de México, Ciudad de México 04510, México; willy_meza_morales@hotmail.com (W.M.-M.); yfar30@hotmail.com (Y.A.-R.); leidyslaura92@gmail.com (L.L.P.-G.); rosario.tavera@gmail.com (R.T.-H.); mtrapan@yahoo.com.mx (M.T.R.-A.); toscano@unam.mx (R.A.T); rubens@unam.mx (R.S.-O.)

* Correspondence: marco.obregon@zaragoza.unam.mx (M.A.O.-M.); habib@unam.mx (R.G.E.); Tel.: +52-55562-24404

Index

Table S1. Crystallographic data of complexes 2 and 3. 1

Fig S1. The four main conformational isomers of DiMeOC calculated at MMFF94**s** level, together with their relative stability in kJ/mol and population in parenthesis........ 2

Fig S2. ^{1}H NMR spectrum of DiMeOC (500 MHz and DMSO-d_6). 3

Fig S3. ^{13}C NMR spectrum of DiMeOC (125 MHz and DMSO-d_6). 4

Fig S4. HSQC NMR spectrum of DiMeOC (500 MHz and DMSO-d_6). 5

Fig S5. HMBC NMR spectrum of DiMeOC (500 MHz and DMSO-d_6). 6

Fig S6. ROESY NMR spectrum of DiMeOC (500 MHz and DMSO-d_6)........ 7

Fig S7. 1H NMR spectrum of DiMeOC-Ga (400 MHz and DMSO-d6). 8

Fig S8. ^{13}C NMR spectrum of DiMeOC-Ga (100 MHz and DMSO-d_6)........ 9

Fig S9. HSQC NMR spectrum of DiMeOC-Ga (400 MHz and DMSO-d_6). 10

Fig S10. HMBC NMR spectrum of DiMeOC-Ga (400 MHz and DMSO-d_6). 11

Fig S11. 1H NMR spectrum of DiMeOC-In (400 MHz and DMSO-d6)........ 12

Fig S12. ^{13}C NMR spectrum of DiMeOC-In (100 MHz and DMSO-d_6). 13

Fig S13. HSQC NMR spectrum of DiMeOC-In (400 MHz and DMSO-d_6). 14

Fig S14. HMBC NMR spectrum of DiMeOC-In (400 MHz and DMSO-d_6)........ 15

Fig S15. 150 MHz 13C ssNMR spectrum of DiMeOC. 16

Fig S16. 150 MHz ^{13}C ssNMR spectrum of DiMeOC-Ga. 17

Fig S17 150 MHz 13C ssNMR spectrum of DiMeOC-In 18

Fig S18. IR Spectrum of DiMeOC........ 19

Fig S19. IR Spectrum of DiMeOC-Ga. 20

Fig S20. IR Spectrum of DiMeOC-In. 21

Fig S21. Mass Spectrum of DiMeOC. 22

Fig S22. Mass Spectrum of DiMeOC-Ga........ 23

Fig S23. Mass Spectrum of DiMeOC-In 24

Fig S24. Mass Spectrum of DiMeOC-Ga........ 25

Fig S25. Mass Spectrum of DiMeOC-In. 26

Fig S26. Elemental analysis of complexes **2** and **3**. 27

Fig S27. UV-Vis of DiMeOC. 28

Fig S28. UV-Vis of DiMeOC-Ga. 29

Fig S29. UV-Vis of DiMeOC-In. 30

Fig S30. Emission spectrum of DiMeOC. 31

Fig S31. Emission spectrum of DiMeOC-Ga. 32
Fig S32. Emission spectrum of DiMeOC-In. 33
Fig S33. UV-Vis DiMeOC in water and 1% of DMSO (For 5 days). 34
Fig S34. UV-Vis DiMeOC-Ga in water and 1% of DMSO (For 5 days). 35
Fig S35. ^{1}H NMR DiMeOC in solution (DMSO-d_6, for 48 hours).. 36
Fig S36. ^{1}H NMR DiMeOC-Ga in solution (DMSO-d_6, for 48 hours). 37
Fig S37. ^{1}H NMR DiMeOC-In in solution (DMSO-d_6, for 48 hours). 38

Table S1. Crystallographic data of complexes 2 and 3.

Complex	2	3
Empirical formula	$C_{52}H_{60}N_2O_{14}Ga_2Cl_4$	$C_{78}H_{90}InN_3O_{21}$
Formula weight	1218.26	1520.34
Temperature (K)	298(2)	150(2)
Crystal system	Triclinic	triclinic
Space group	P-1	P-1
a (Å)	8.1680(8)	14.2026(7)
b (Å)	14.0425(15)	16.4738(8)
c (Å)	26.189(3)	17.8136(8)
α (°)	78.121(3)	92.7110(10)
β (°)	81.030(3)	106.8320(10)
γ (°)	83.967(3)	110.7890(10)
Volume ($Å^3$)	2895.3(5)	3676.7(3)
Z	2	2
ρ_{calc}(g /cm^3)	1.397	1.373
μ (mm^{-1})	1.177	0.398
F(000)	1256.0	1592.0
Crystal size (mm^3)	0.412 × 0.047 × 0.045	0.415 × 0.227 × 0.144
Radiation	MoKα (λ = 0.71073)	MoKα (λ = 0.71073)
2Θ range for data collection (°)	4.776 to 51.604	4.458 to 60.244
Index ranges	-9 ≤ h ≤ 9, -17 ≤ k ≤ 17, -31 ≤ l ≤ 32	-20 ≤ h ≤ 20, -23 ≤ k ≤ 23, -25 ≤ l ≤ 25
Reflections collected	49015	256997
Independent reflections	11003 [R_{int} = 0.1516, R_{sigma} = 0.1640]	21627 [R_{int} = 0.0743, R_{sigma} = 0.0412]
Data/restraints/parameters	11003/676/775	21627/0/946
Goodness-of-fit on F^2	1.001	1.060
Final R indexes [I>=2σ (I)]	R_1 = 0.0707, wR_2 = 0.1352	R_1 = 0.0431, wR_2 = 0.0871
Final R indexes [all data]	R_1 = 0.2205, wR_2 = 0.1893	R_1 = 0.0638, wR_2 = 0.0953
Largest diff. peak/hole (e $Å^{-3}$)	0.59/-0.34	0.68/-0.49

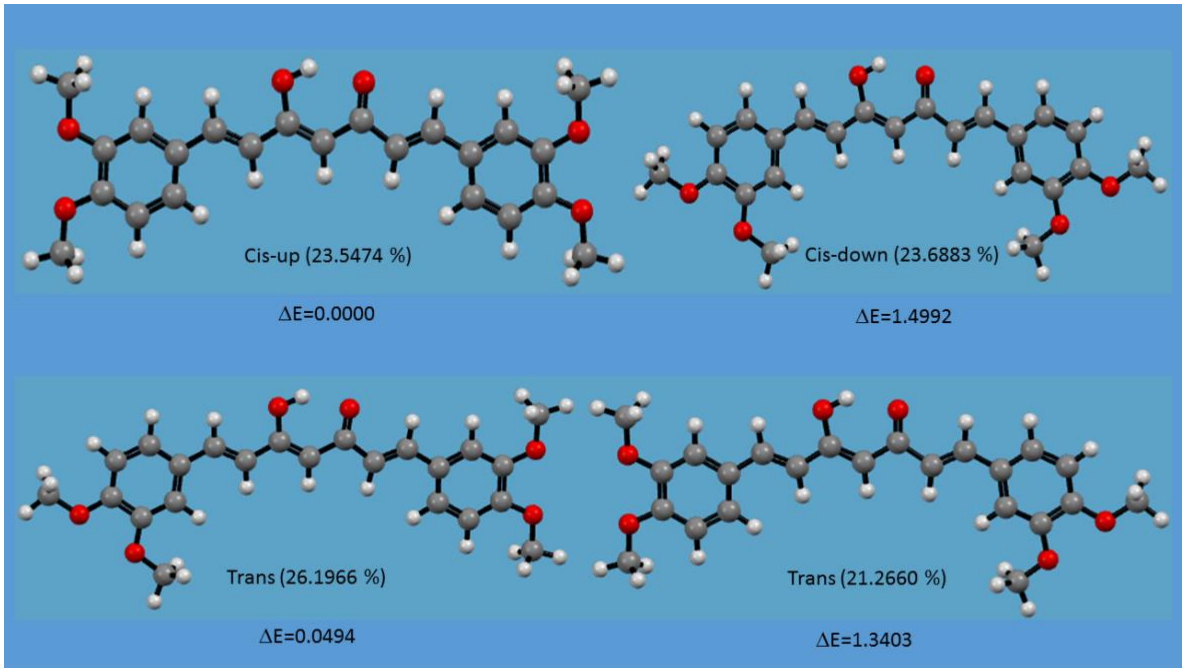

Fig S1. The four main conformational isomers of DiMeOC calculated at MMFF94**s** level, together with their relative stability in kJ/mol and population in parenthesis.

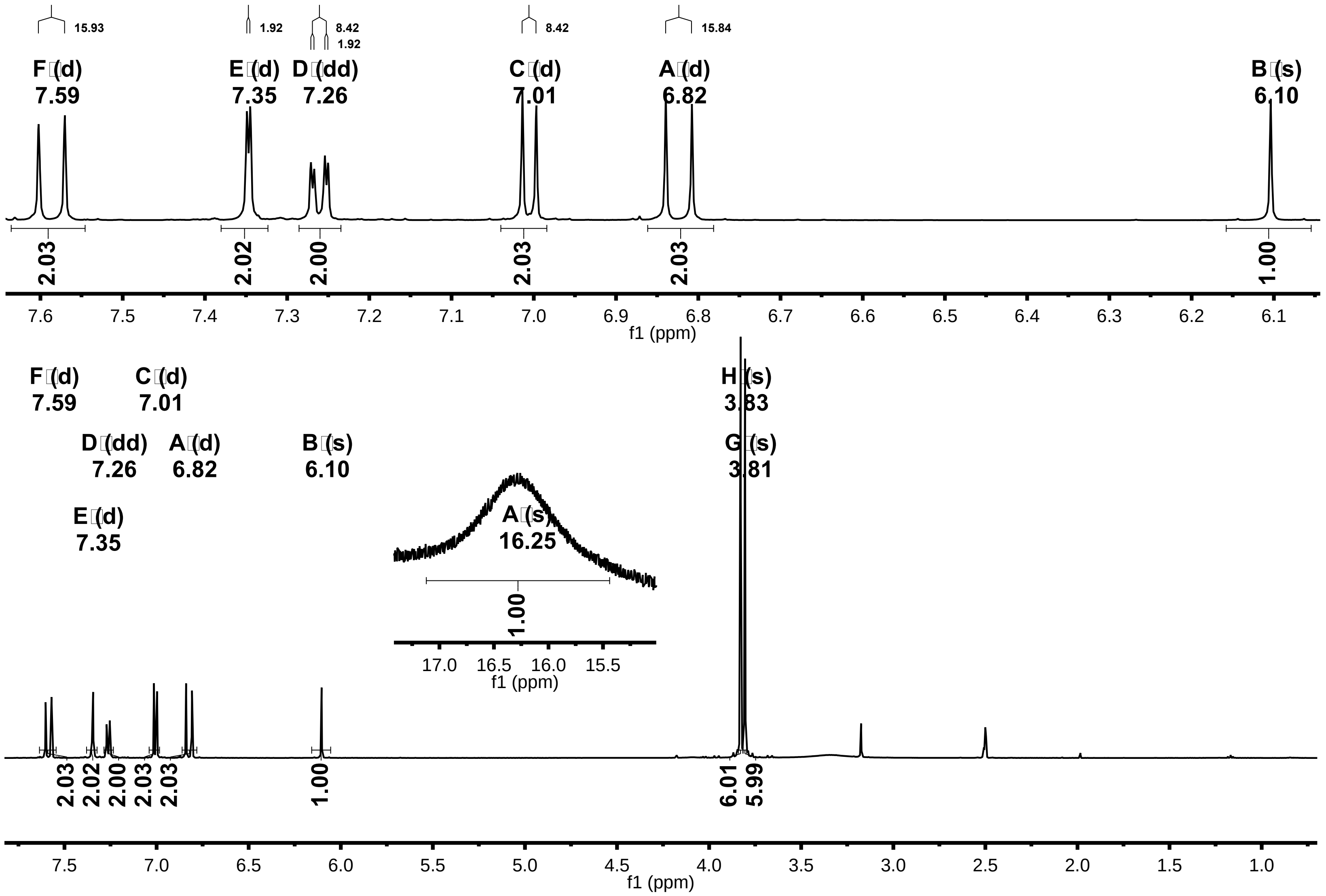

Fig S2. ^{1}H NMR spectrum of DiMeOC (500 MHz and DMSO-d_6).

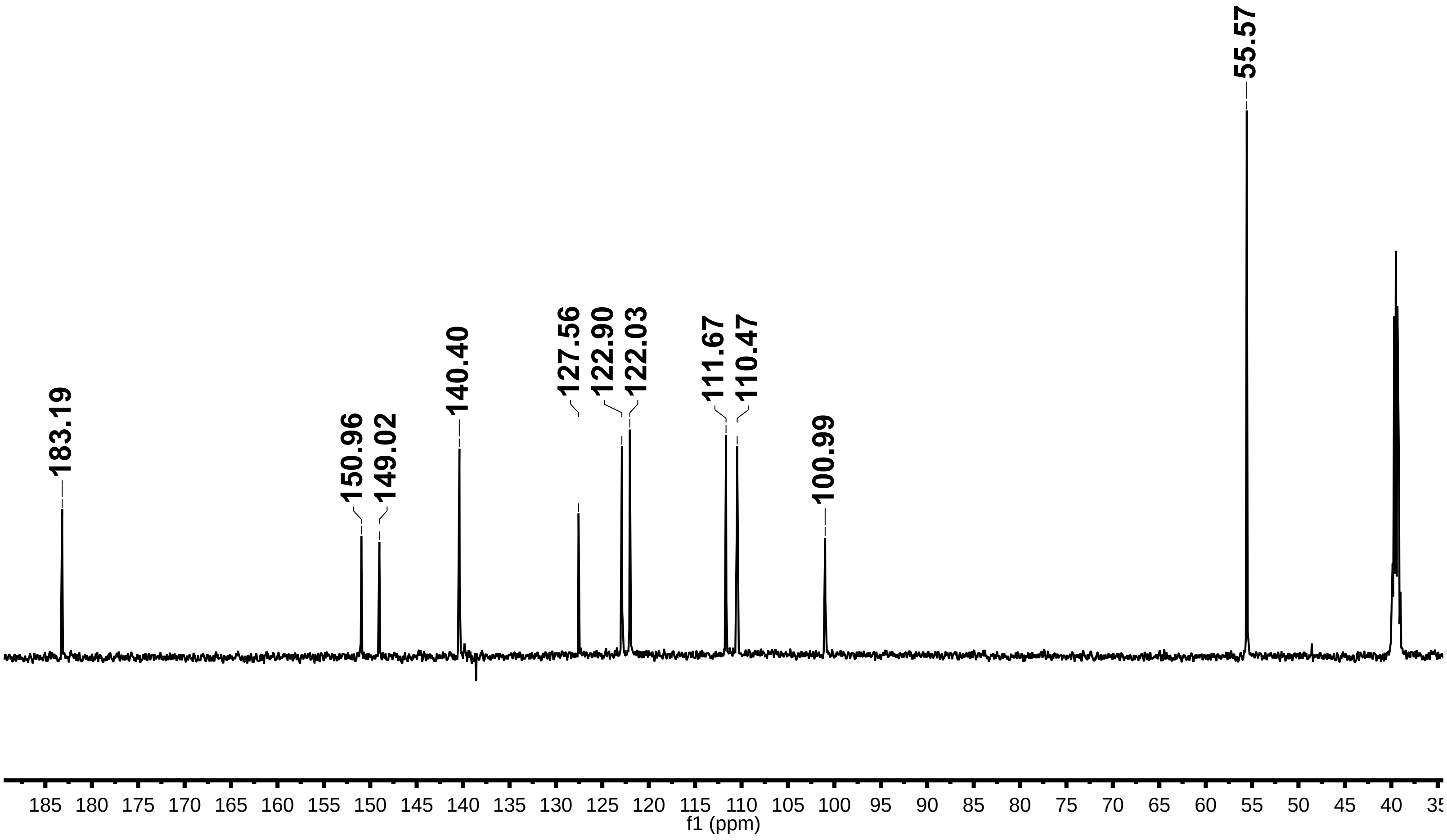

Fig S3. ^{13}C NMR spectrum of DiMeOC (125 MHz and DMSO-d_6).

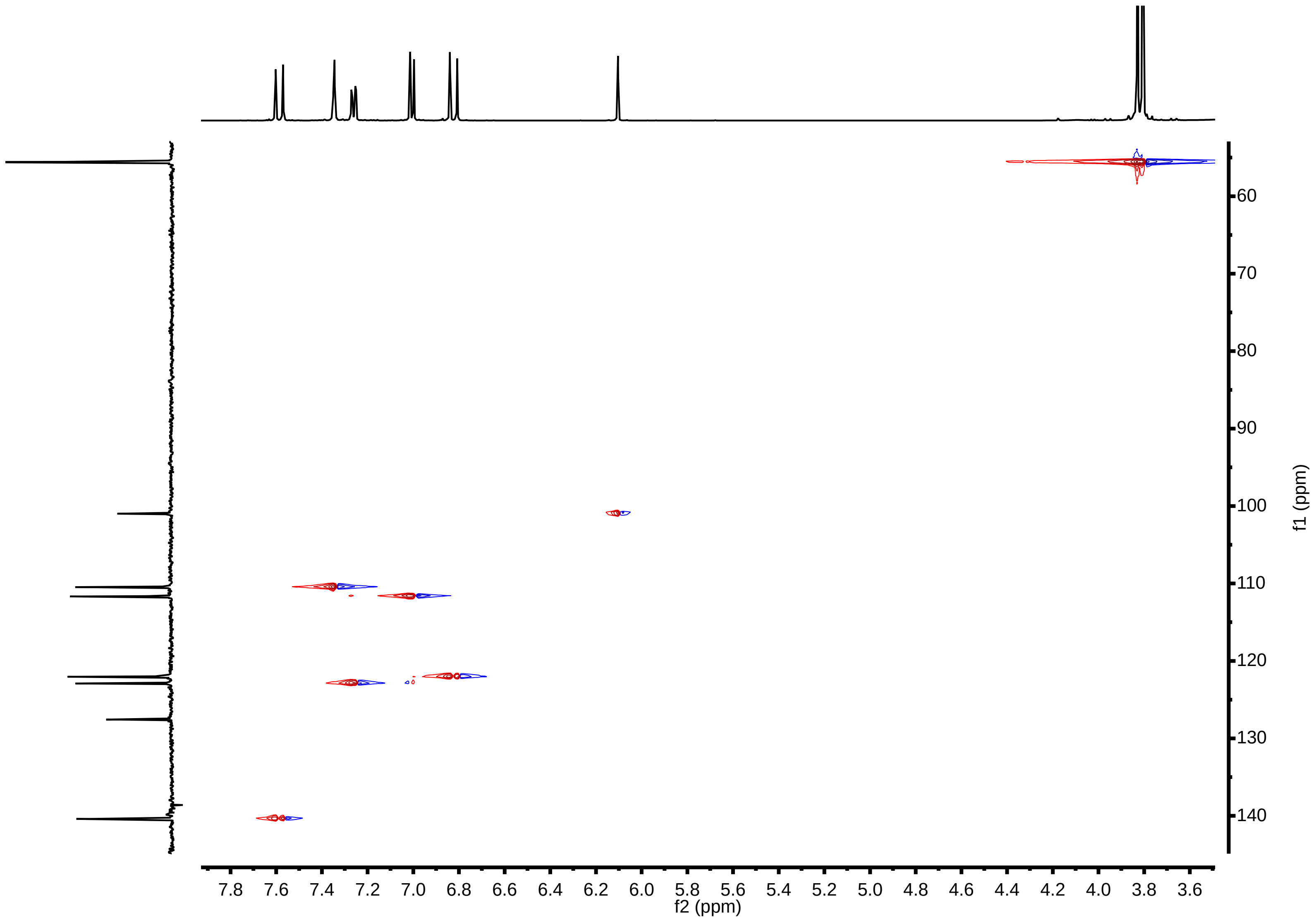

Fig S4. HSQC NMR spectrum of DiMeOC (500 MHz and DMSO-d_6).

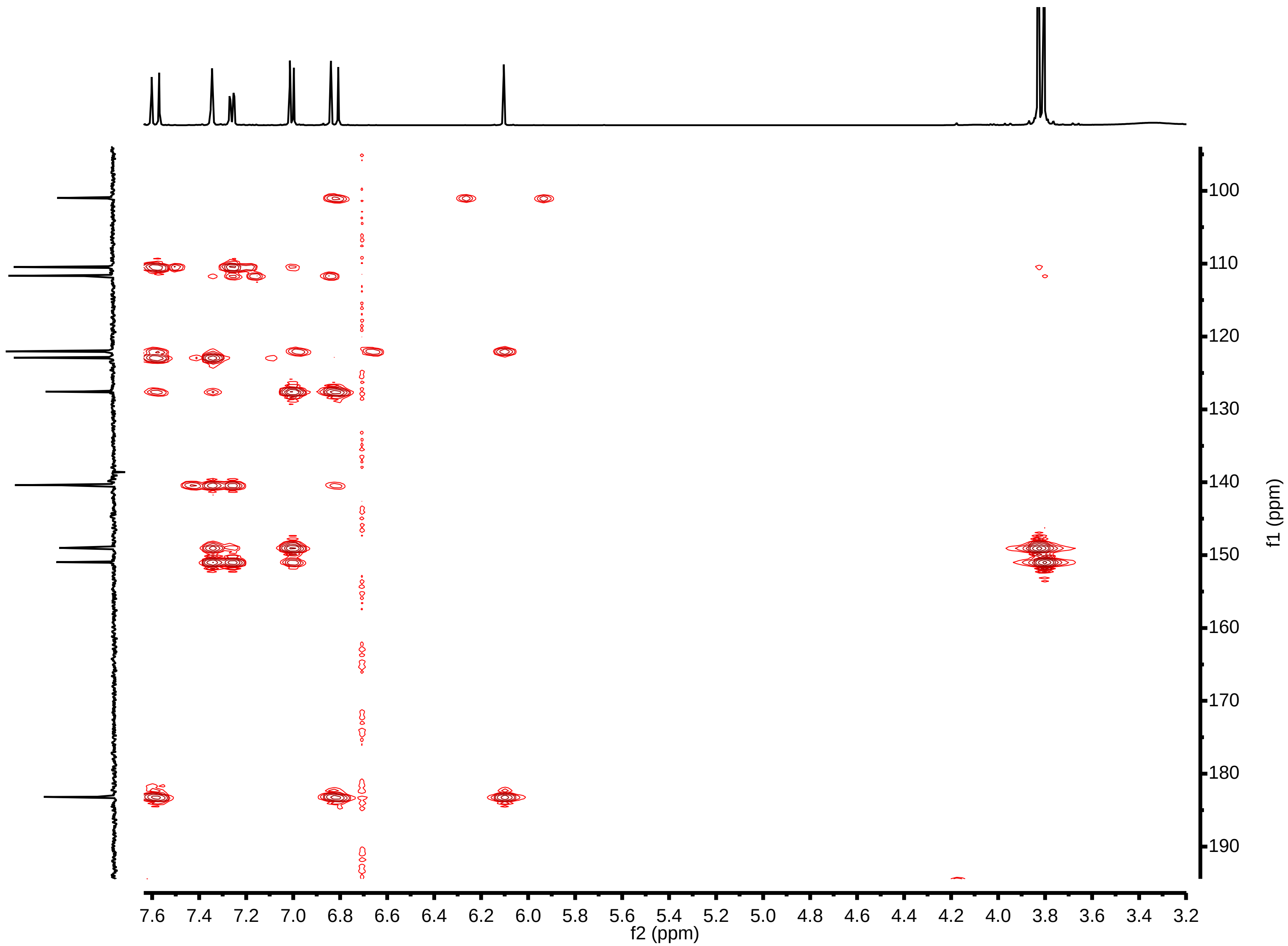

Fig S5. HMBC NMR spectrum of DiMeOC (500 MHz and DMSO-d_6).

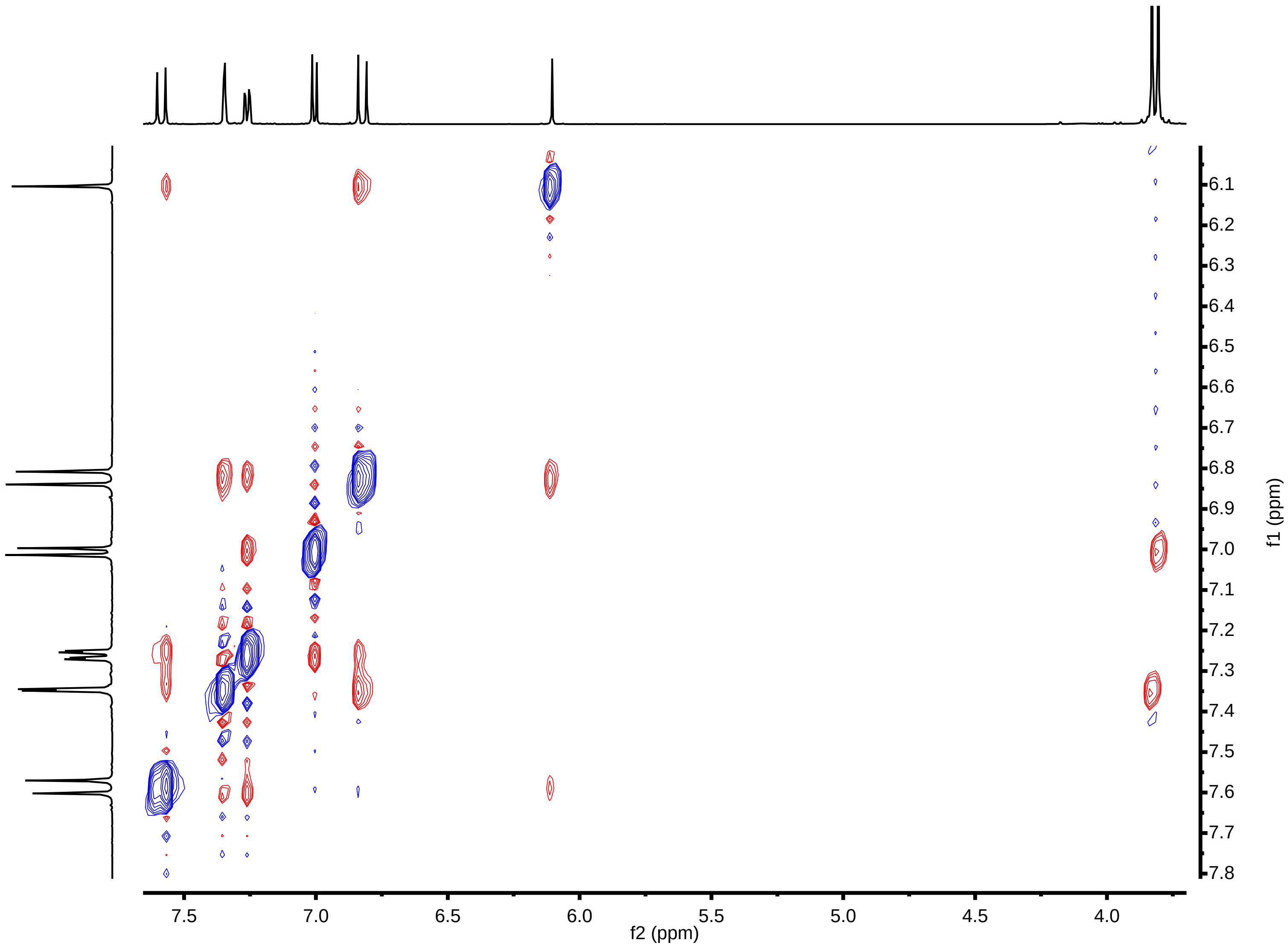

Fig S6. ROESY NMR spectrum of DiMeOC (500 MHz and DMSO-d_6).

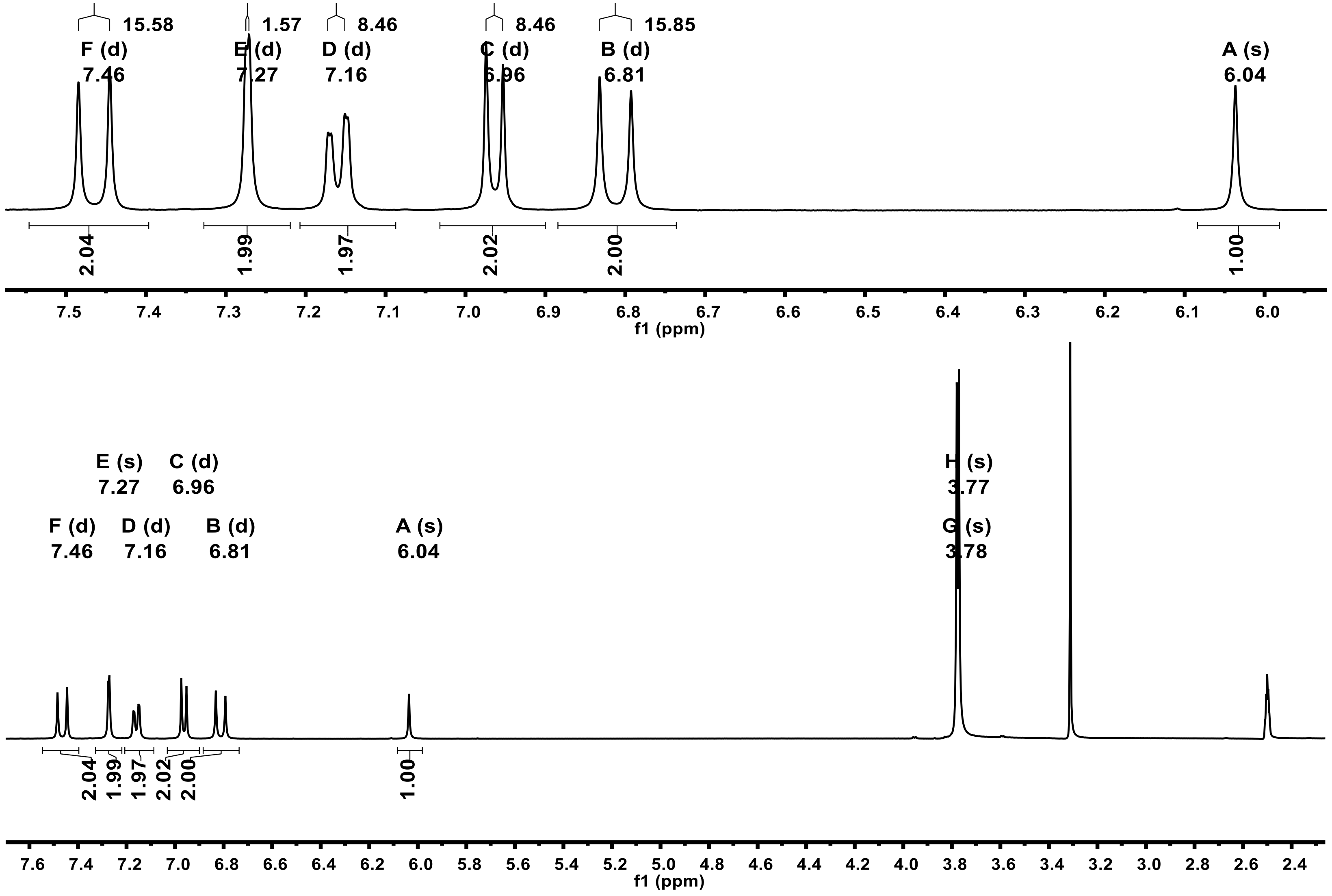

Fig S7. 1H NMR spectrum of DiMeOC-Ga (400 MHz and DMSO-d6).

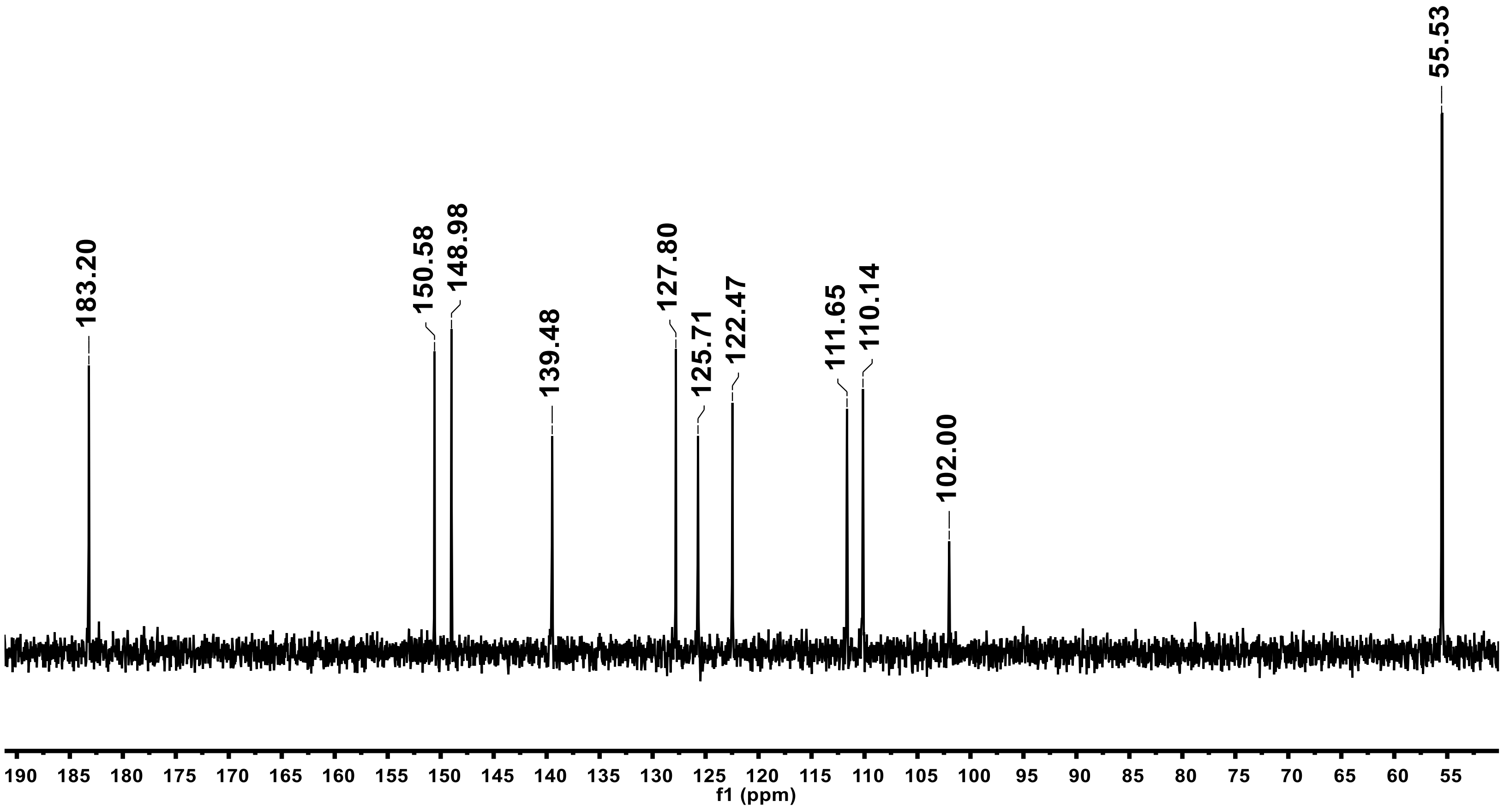

Fig S8. ^{13}C NMR spectrum of DiMeOC-Ga (100 MHz and DMSO-d_6).

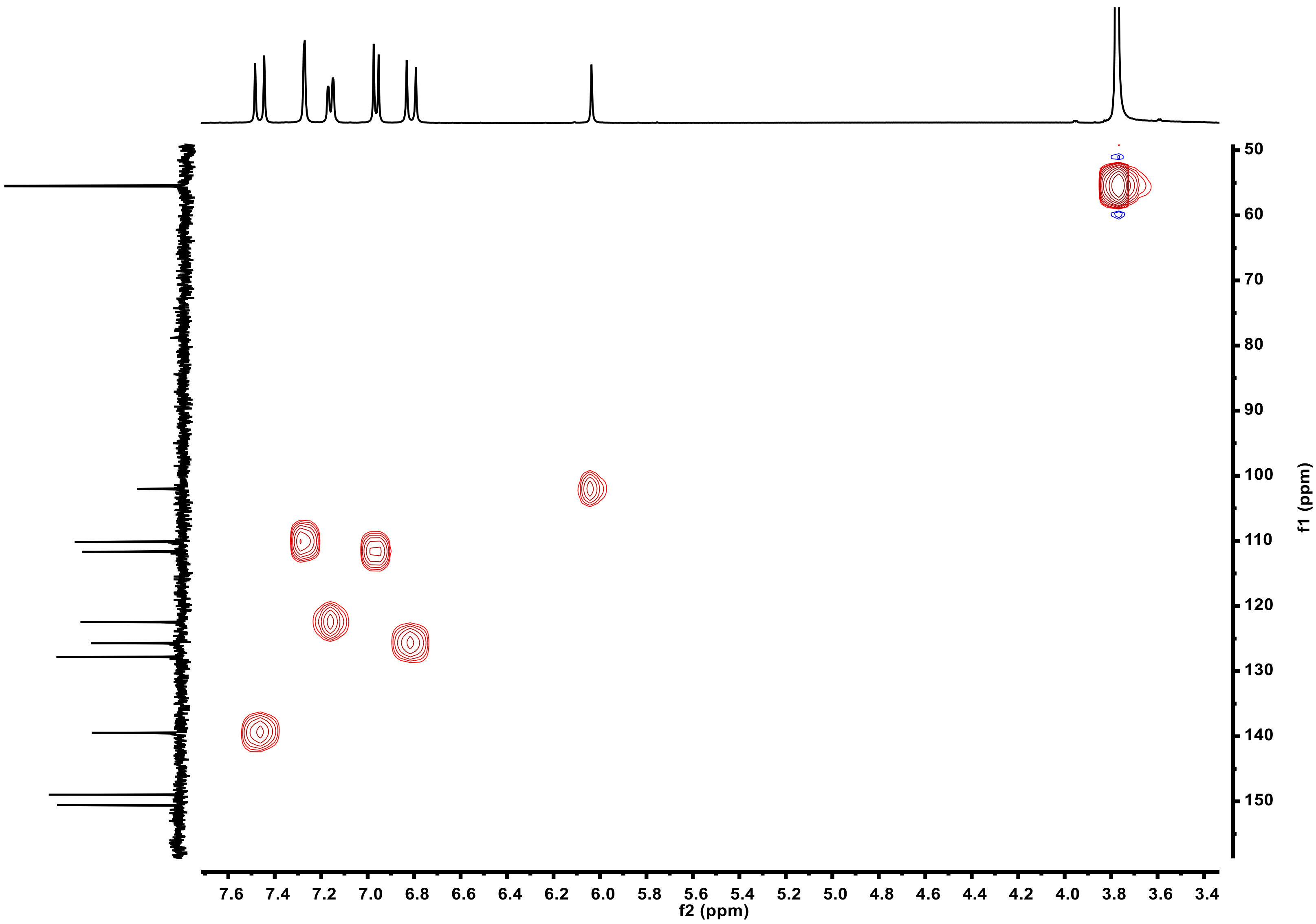

Fig S9. HSQC NMR spectrum of DiMeOC-Ga (400 MHz and DMSO-d_6).

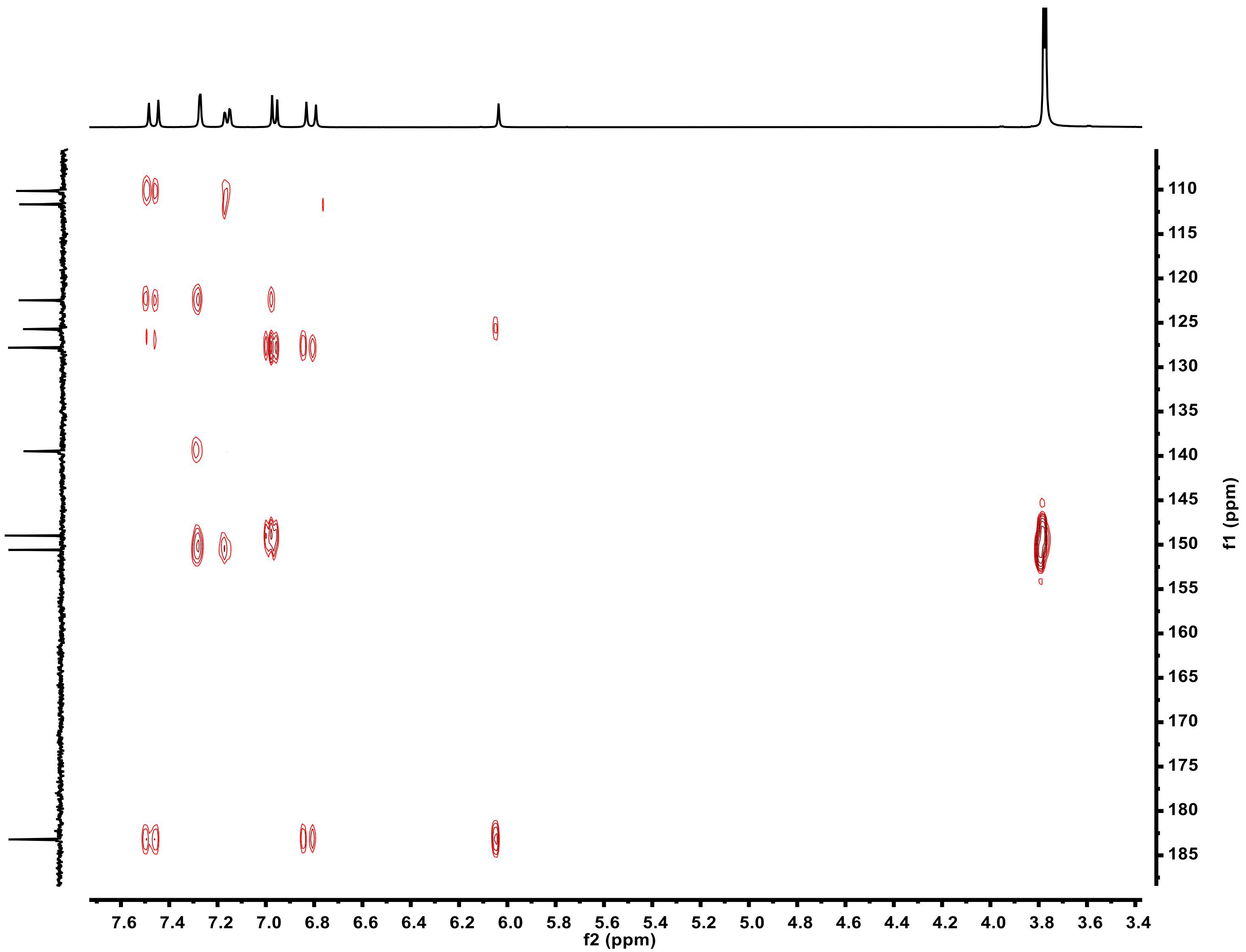

Fig S10. HMBC NMR spectrum of DiMeOC-Ga (400 MHz and DMSO-d_6).

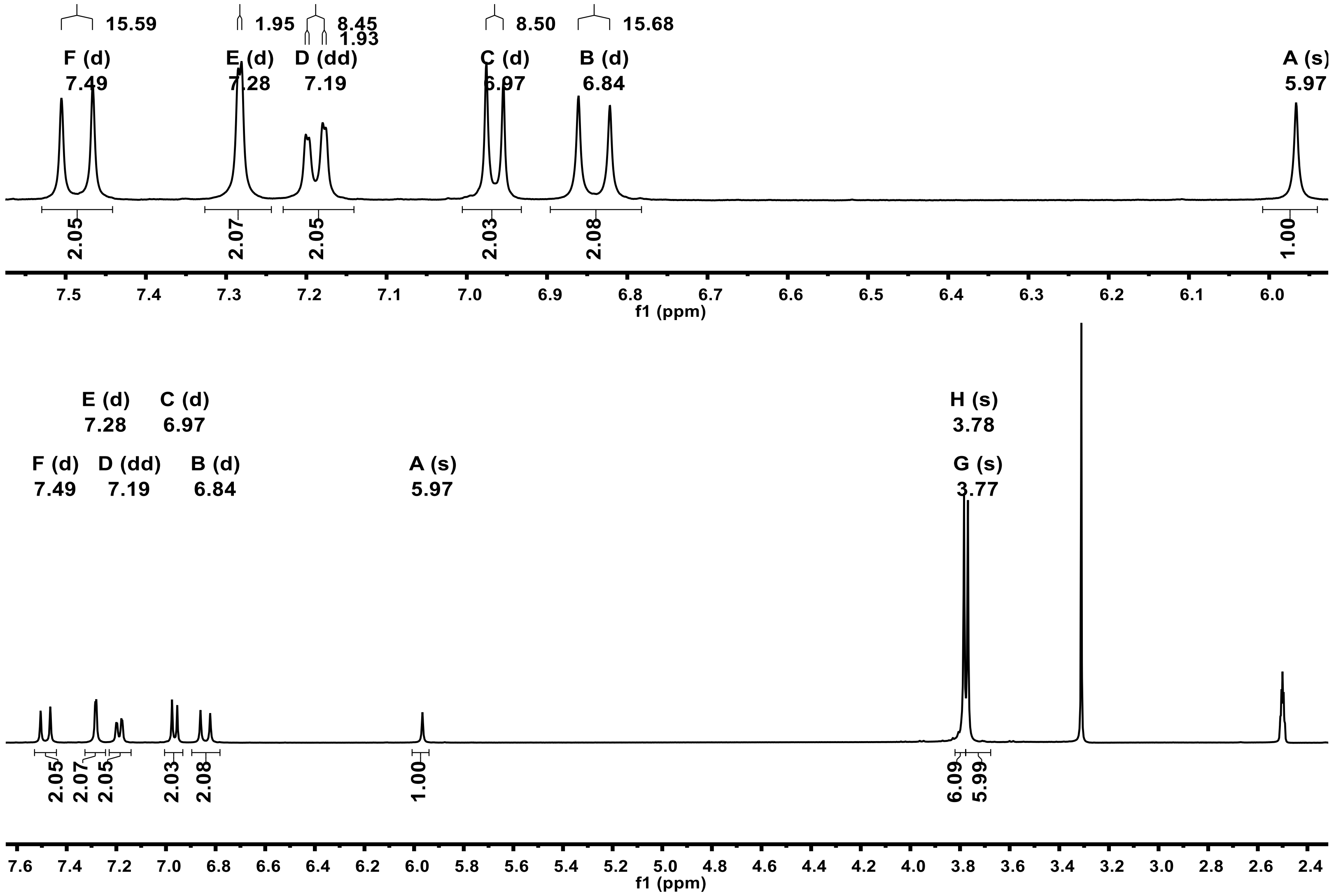

Fig S11. 1H NMR spectrum of DiMeOC-In (400 MHz and DMSO-d6)

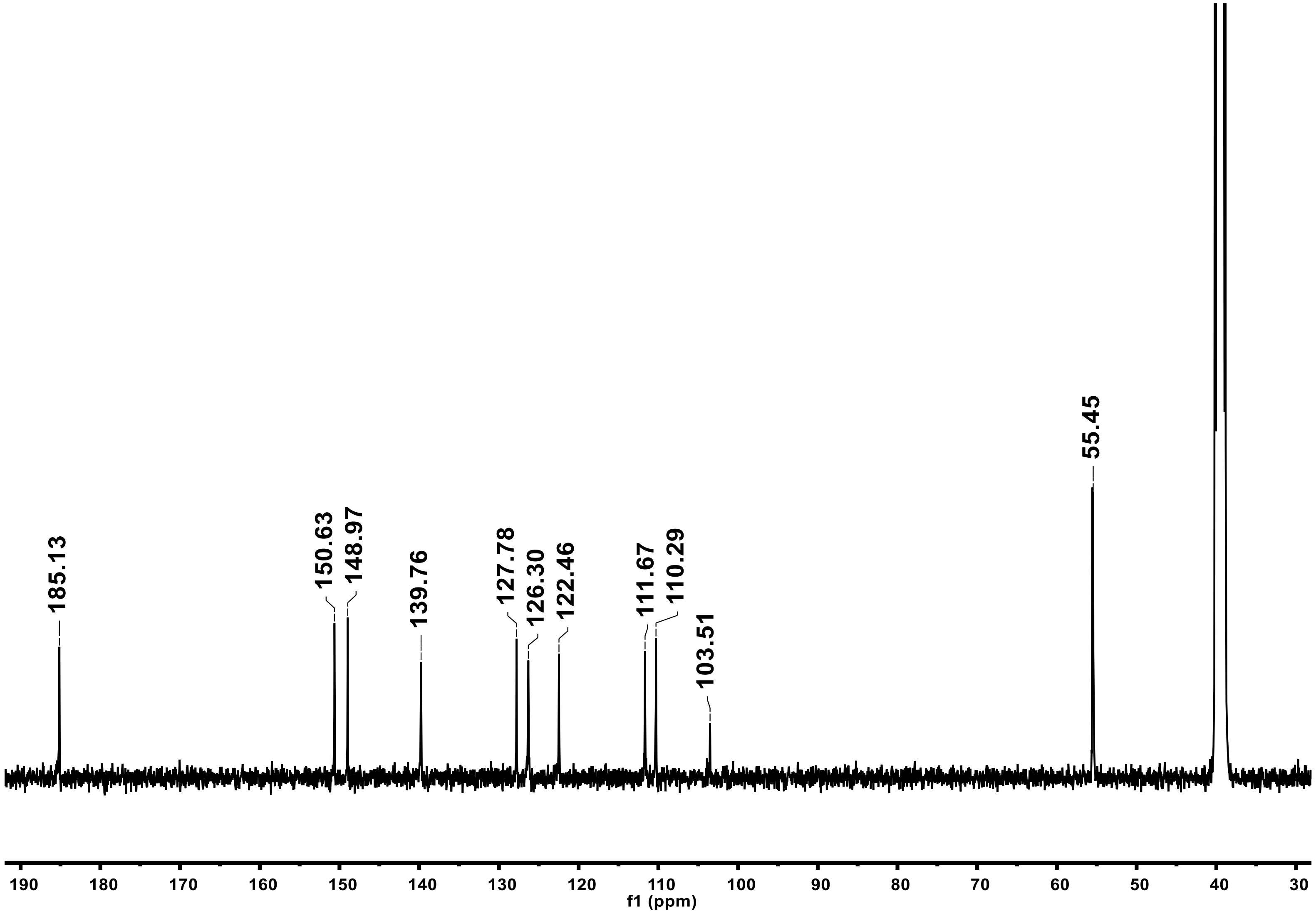

Fig S12. ^{13}C NMR spectrum of DiMeOC-In (100 MHz and DMSO-d_6).

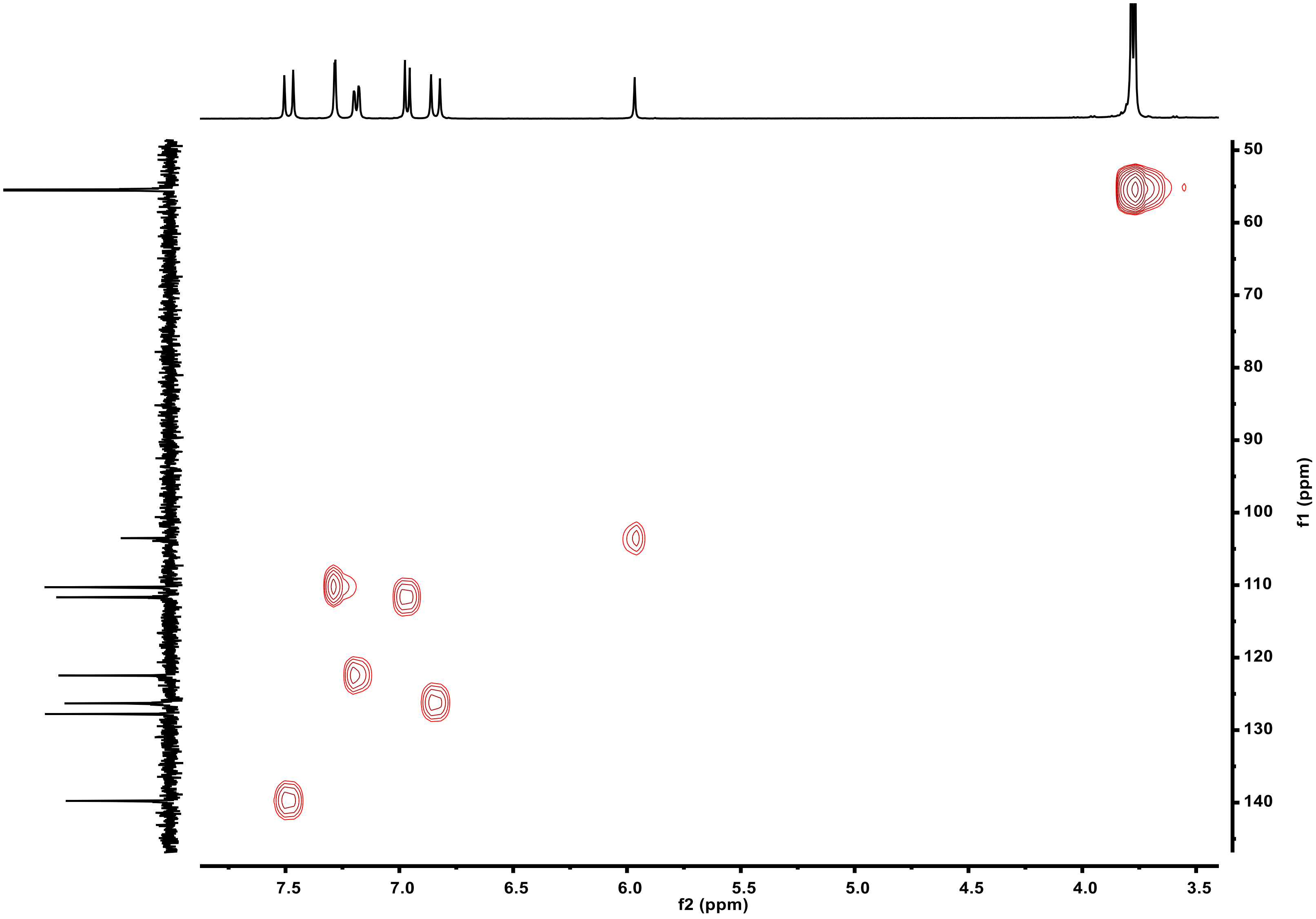

Fig S13. HSQC NMR spectrum of DiMeOC-In (400 MHz and DMSO-d_6).

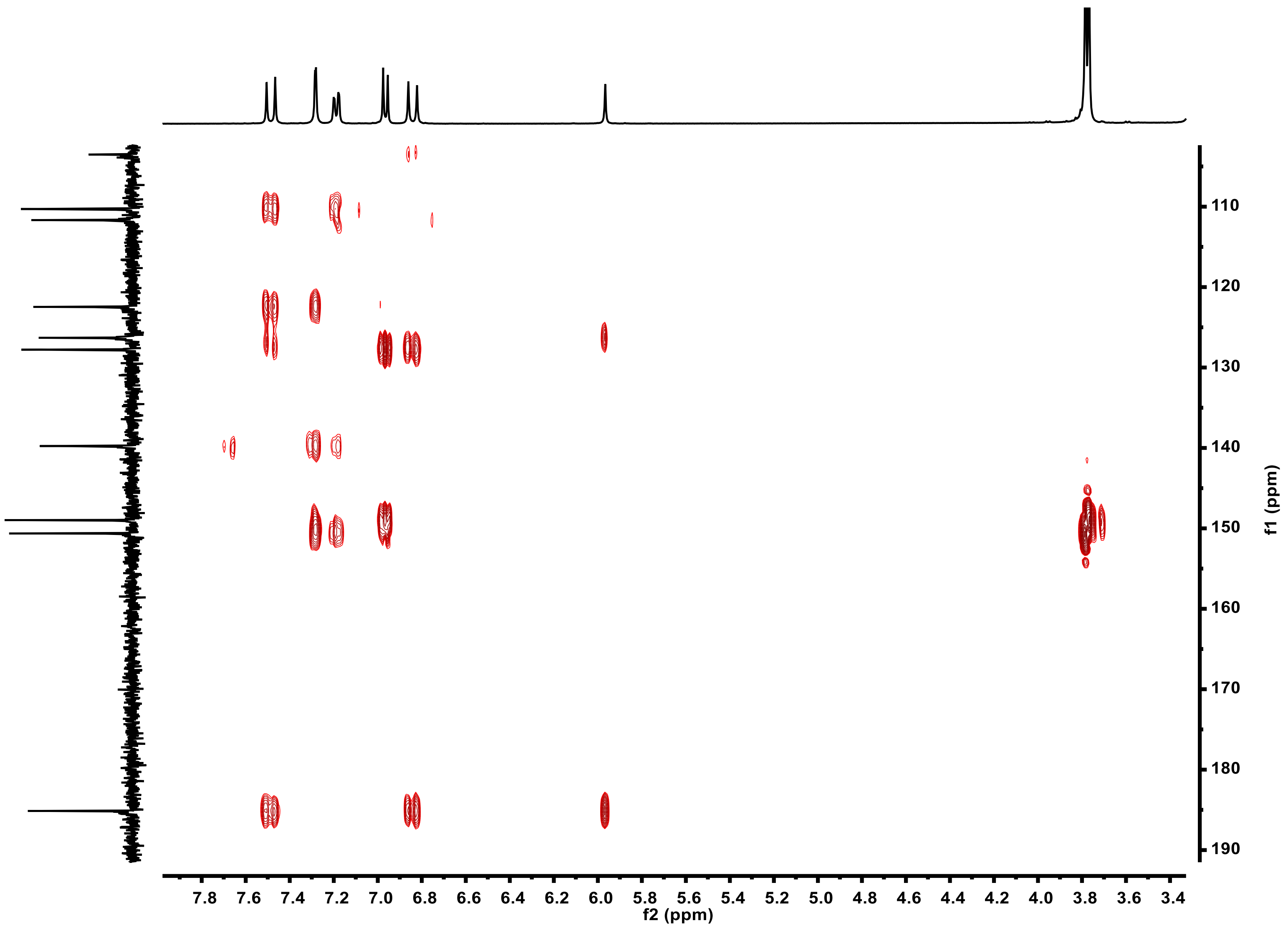

Fig S14. HMBC NMR spectrum of DiMeOC-In (400 MHz and DMSO-d_6).

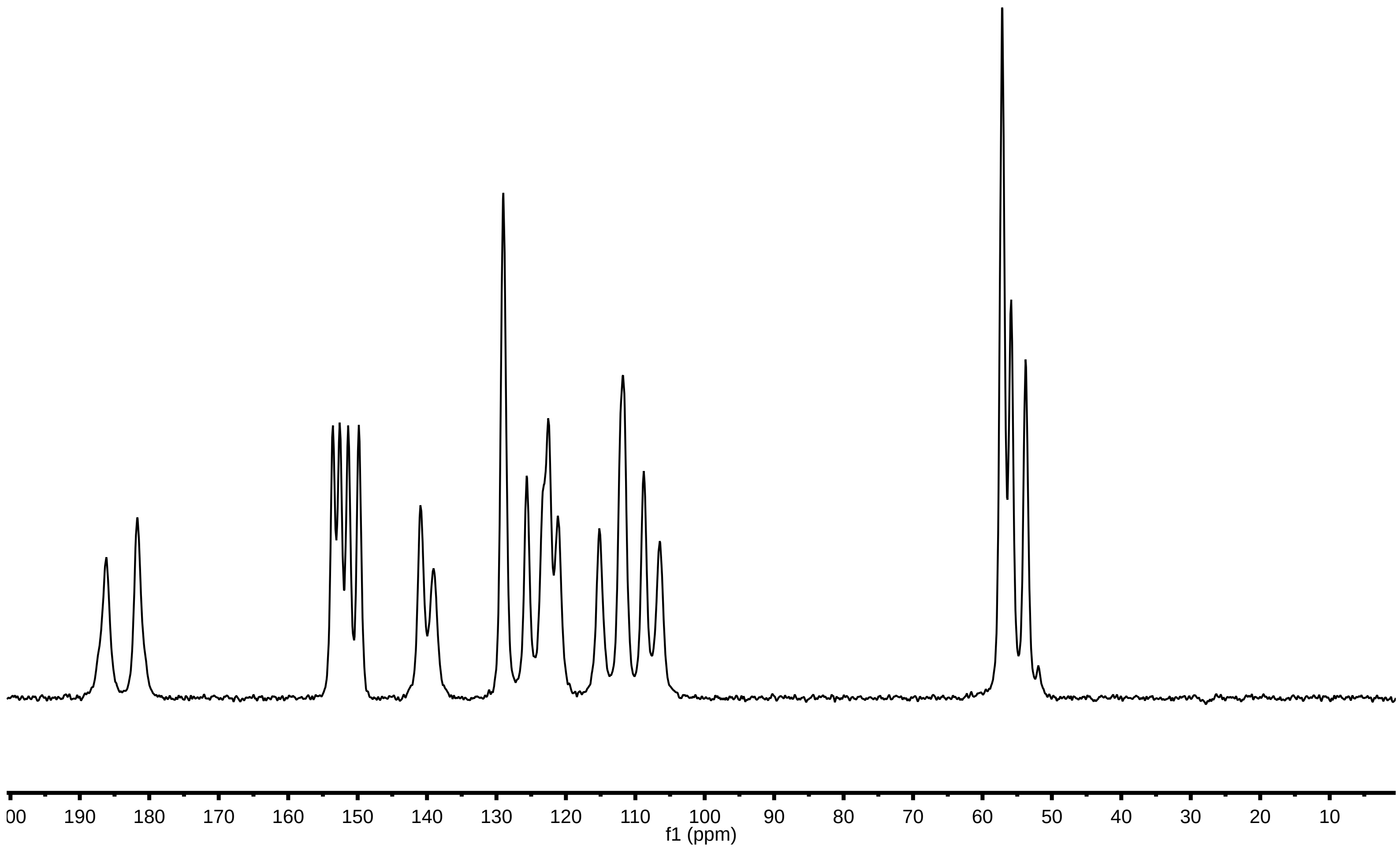

Fig S15. 150 MHz 13C ssNMR spectrum of DiMeOC.

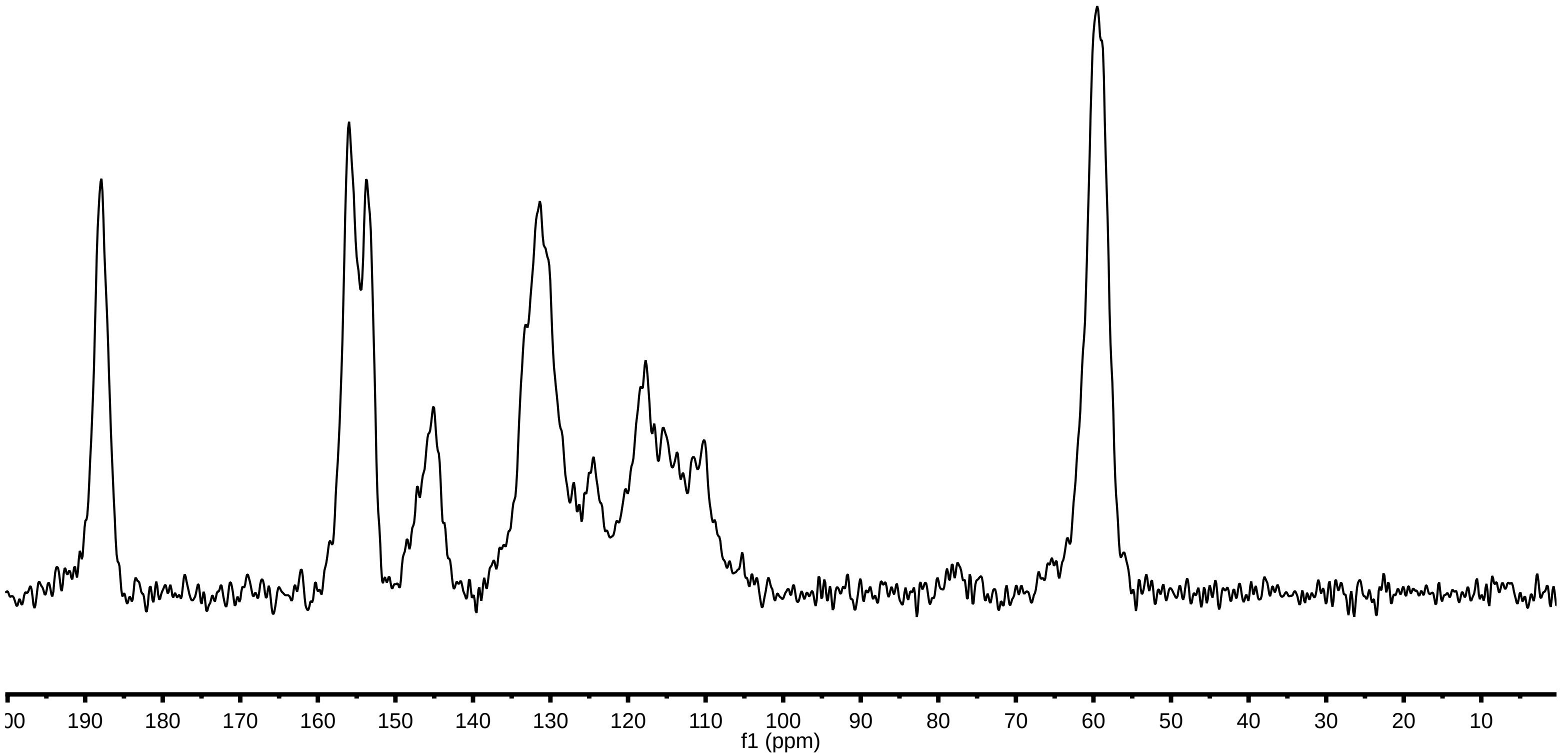

Fig S16. 150 MHz ^{13}C ssNMR spectrum of DiMeOC-Ga.

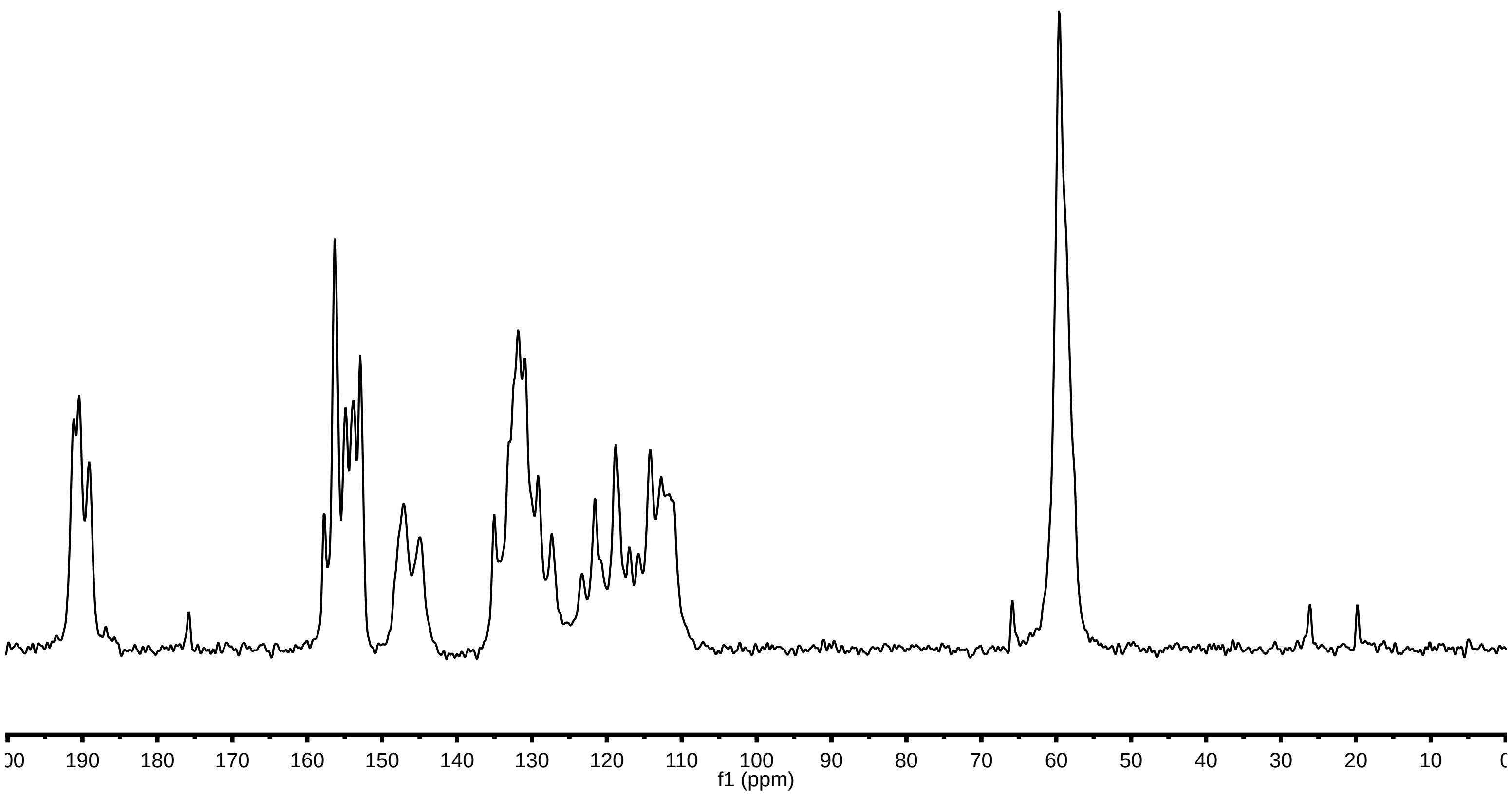

Fig S17 150 MHz 13C ssNMR spectrum of DiMeOC-In

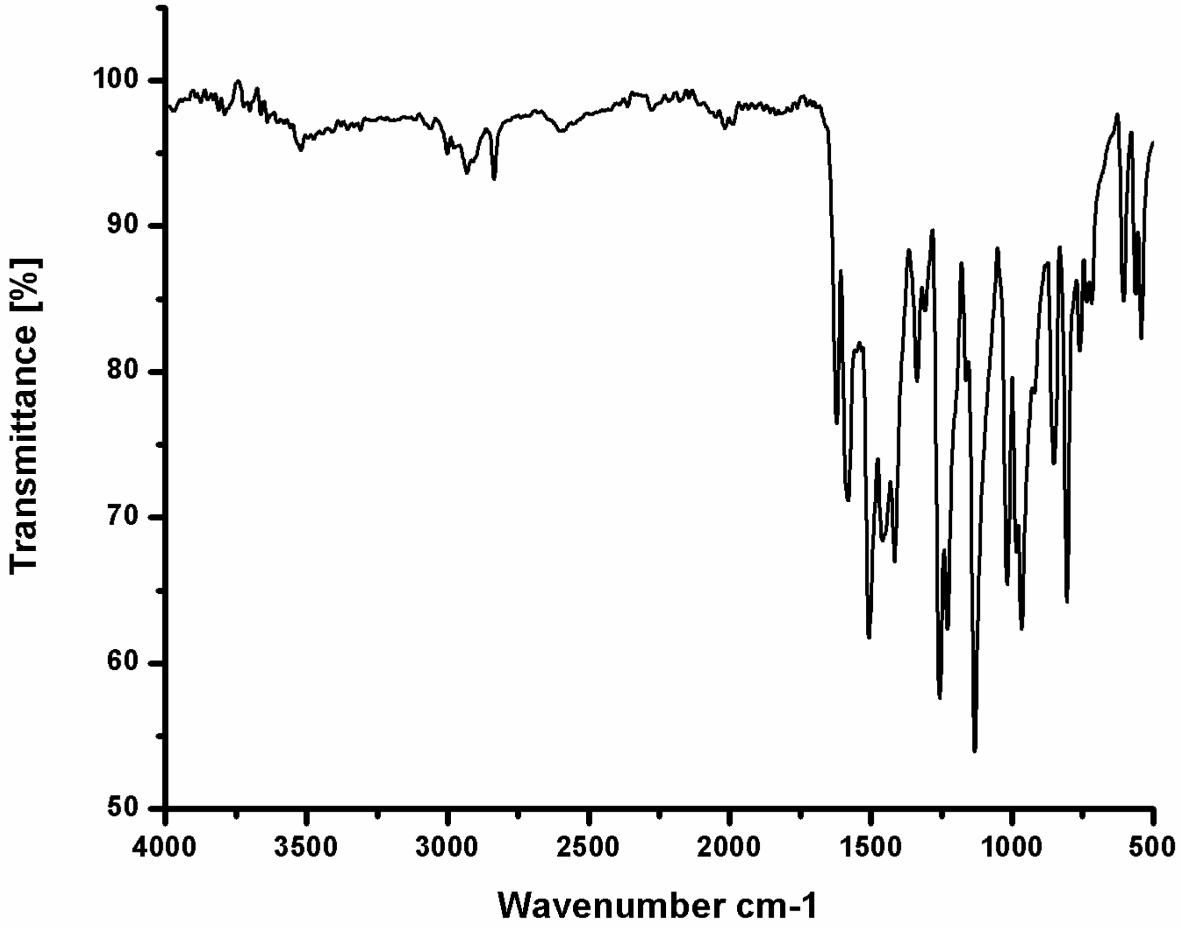

Fig S18. IR Spectrum of DiMeOC.

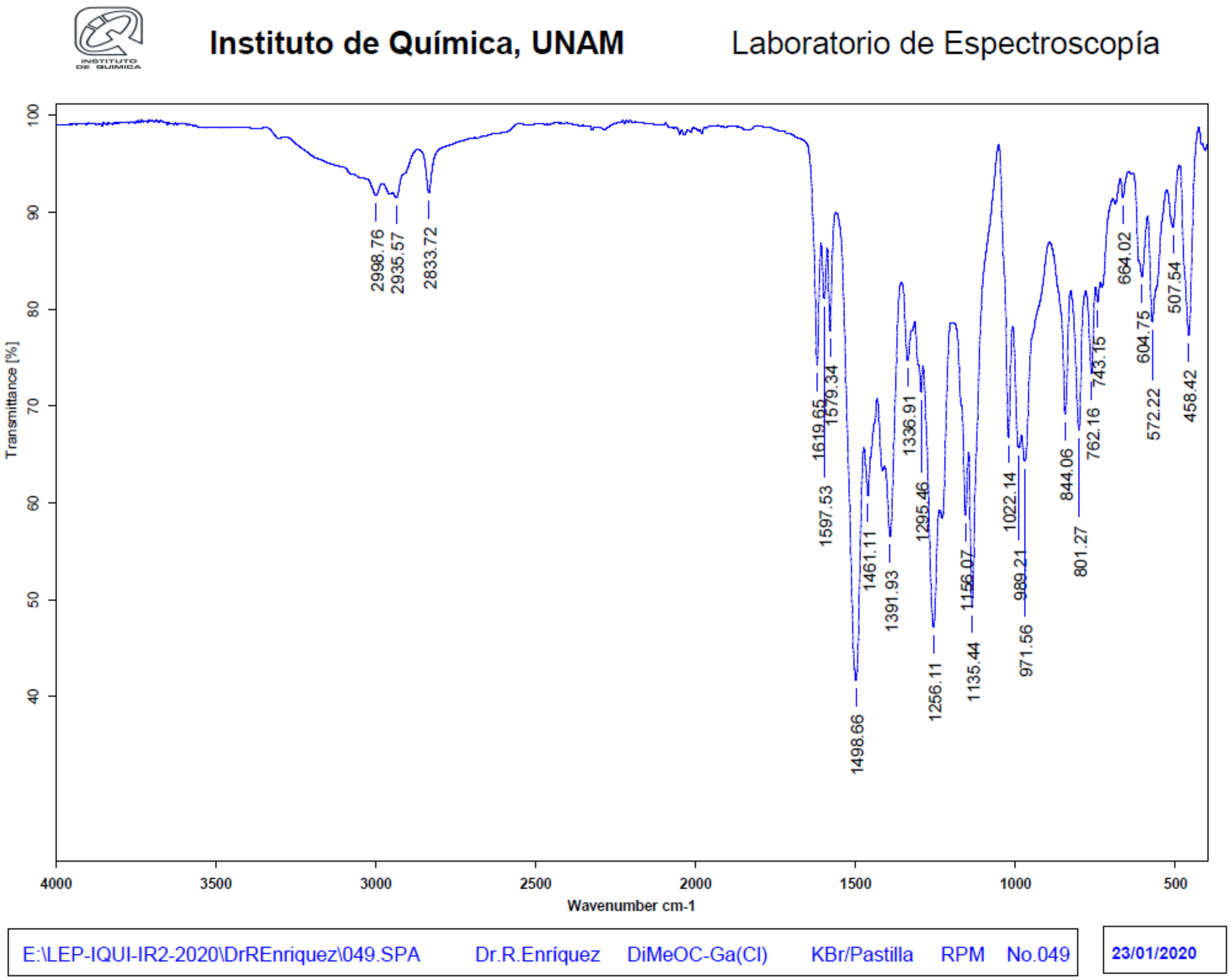

Fig S19. IR Spectrum of DiMeOC-Ga.

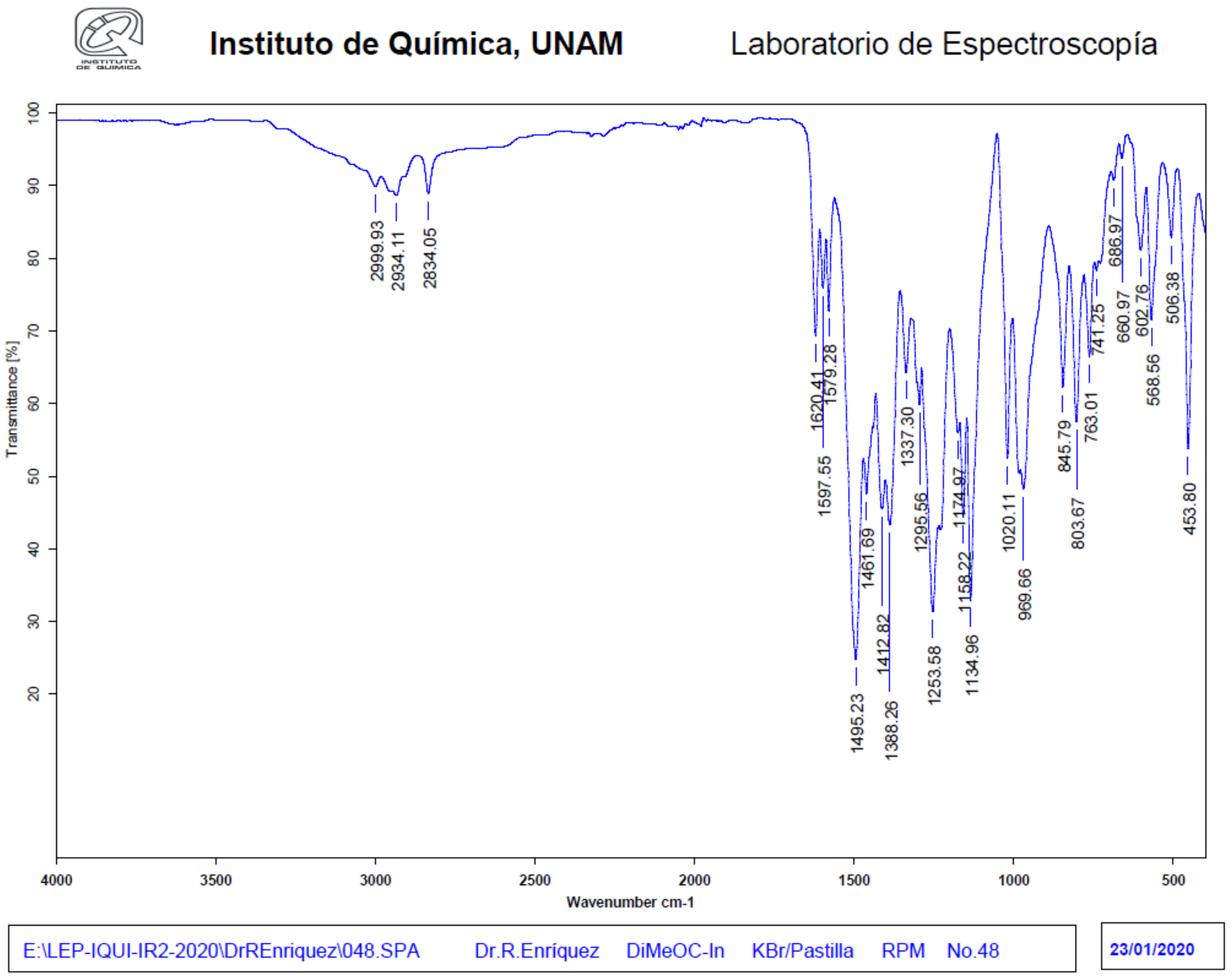

Fig S20. IR Spectrum of DiMeOC-In.

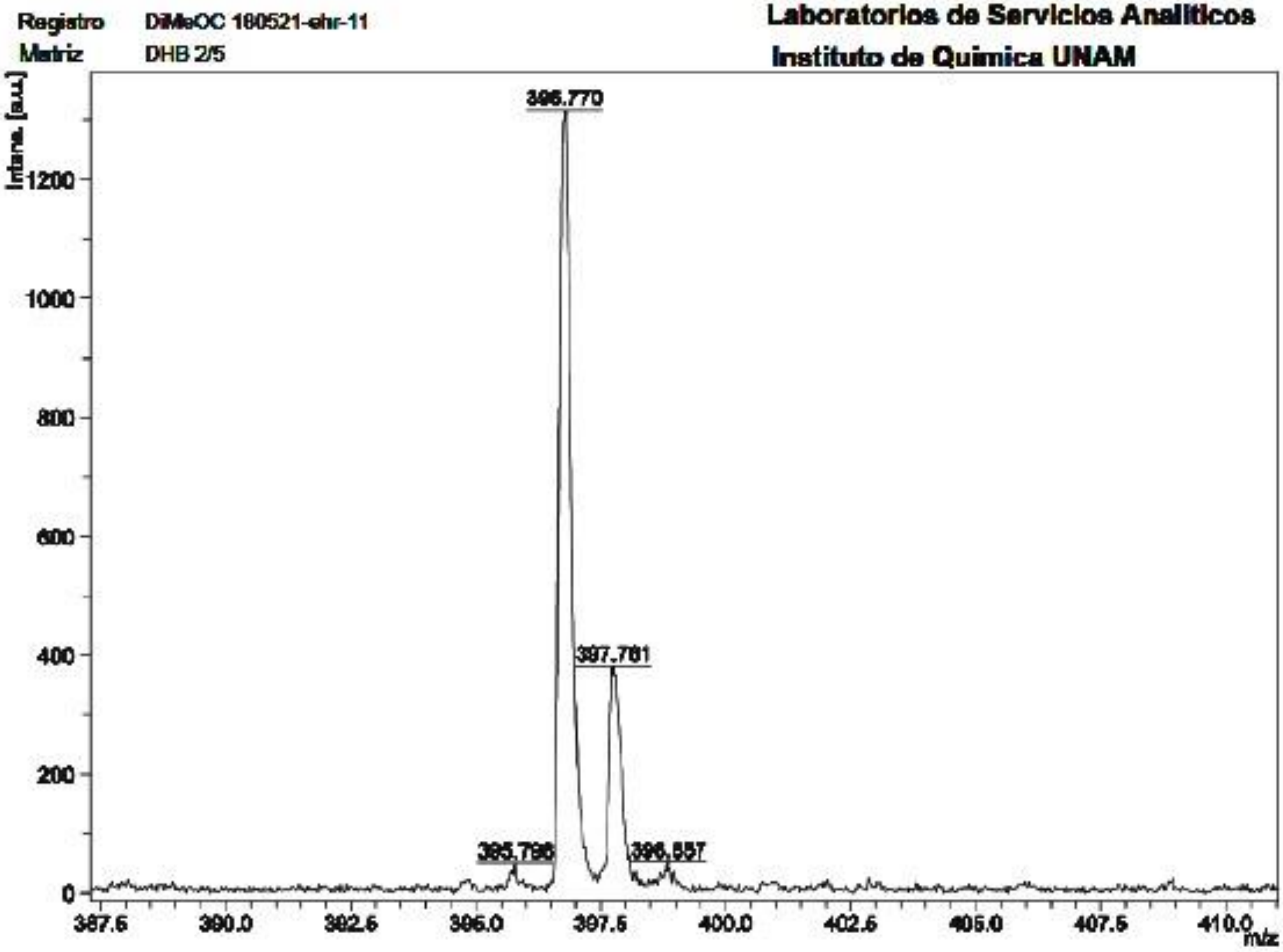

Fig S21. Mass Spectrum of DiMeOC.

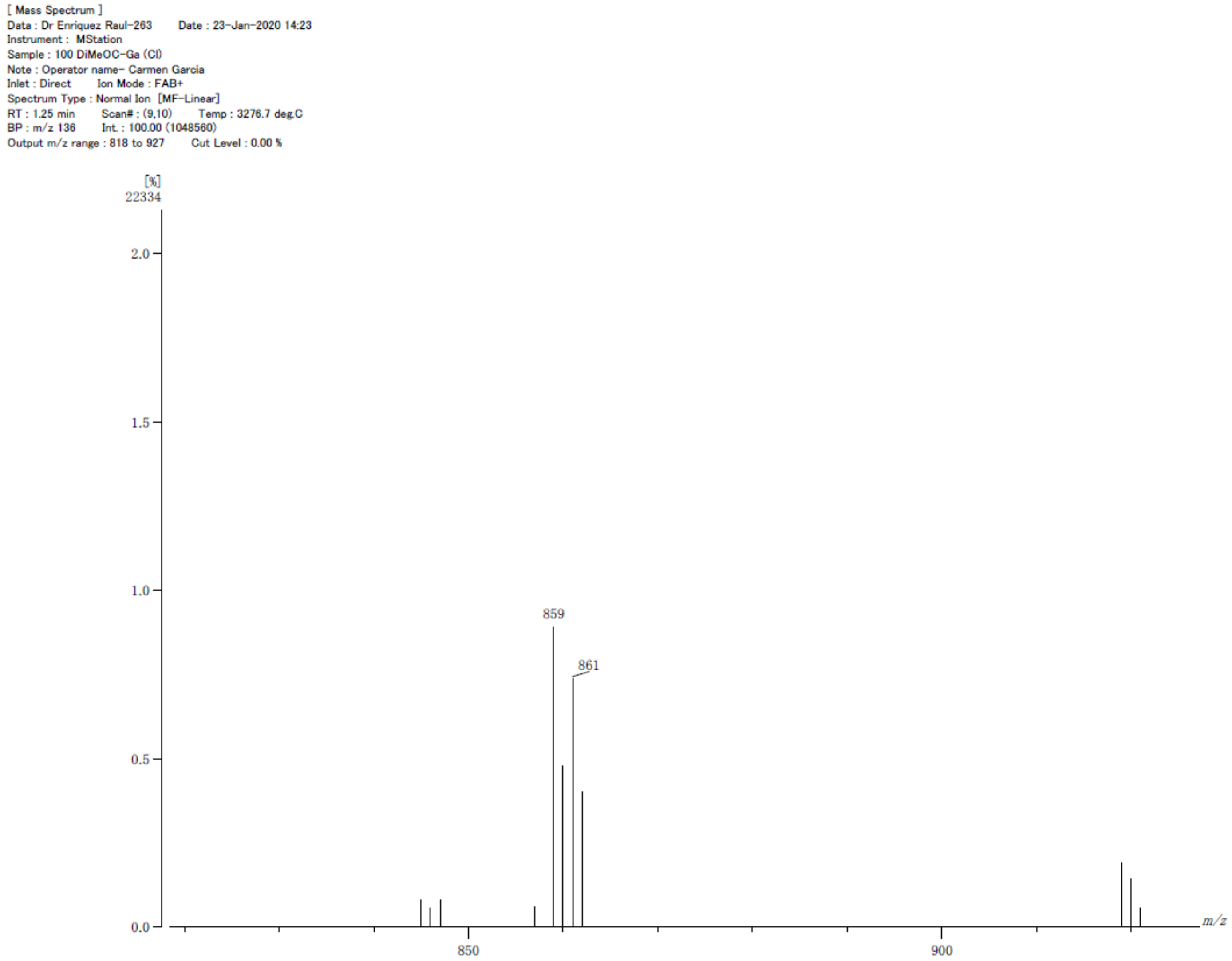

Fig S22. Mass Spectrum of DiMeOC-Ga.

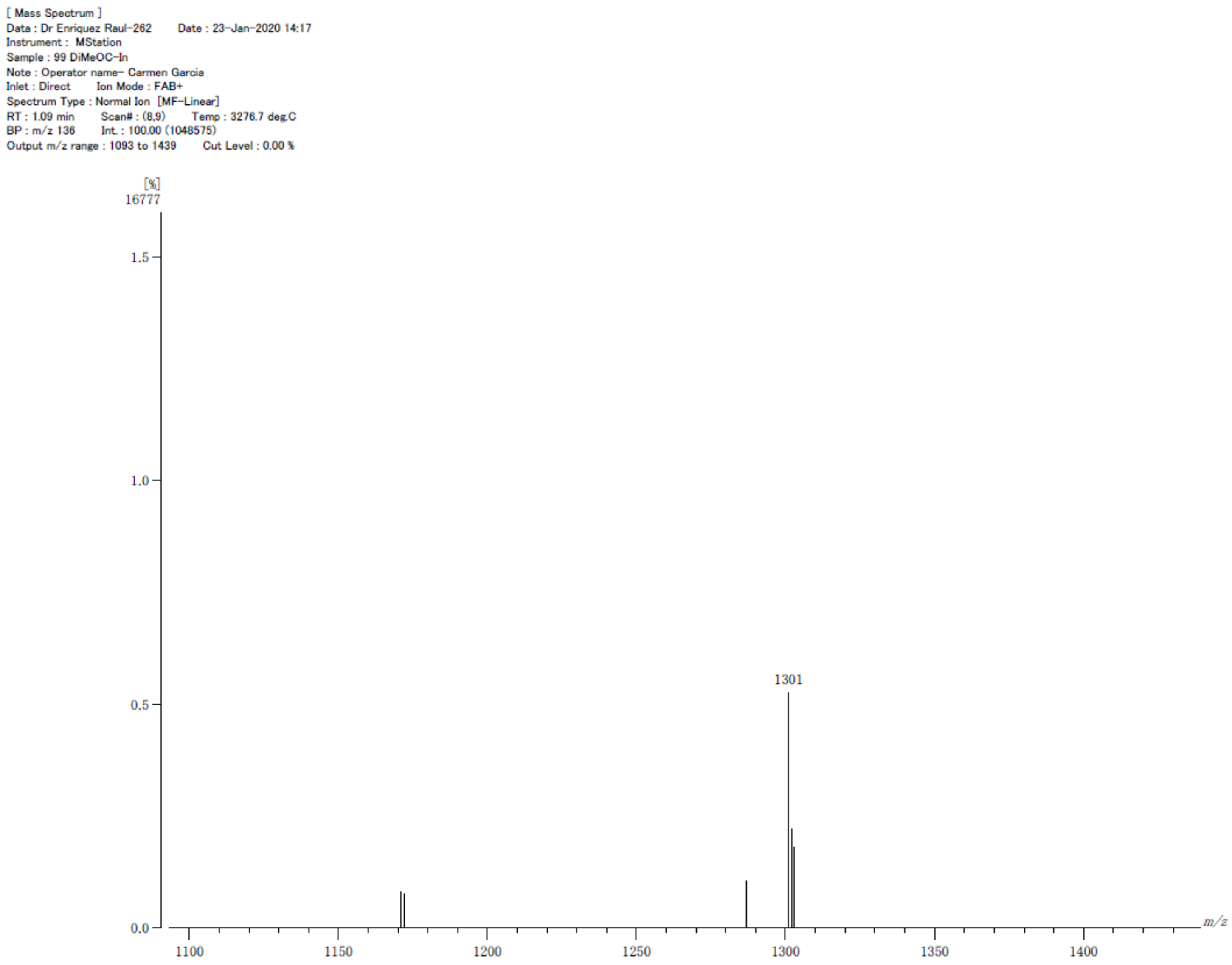

Fig S23. Mass Spectrum of DiMeOC-In.

Data : Dr Enriquez Raul-174 Date : 26-Feb-2020 15:22
Instrument : MStation
Sample : 224 DiMeOC-Ga
Note : Operador: Carmen Garcia
Inlet : Direct Ion Mode : FAB+
RT : 4.83 min Scan# : (72,73)
Elements : C 46/0, H 50/0, O 12/0, Ga 1/0
Mass Tolerance : 1000ppm, 5mmu if m/z > 5
Unsaturation (U.S.) : -0.5 - 30.0

Observed m/z	Int%
859.2254	100.00

	Estimated m/z	Err[ppm / mmu]	U.S.	C	H	O	Ga
1	859.2245	+1.0 / +0.9	24.5	46	46	12	1

Fig S24. Mass Spectrum of DiMeOC-Ga.

Data : Dr Enriquez Raul-150 Date : 06-Feb-2020 10:20
Instrument : MStation
Sample : 0180 DiMeOC-In
Note : Operador: Carmen Garcia
Inlet : Direct Ion Mode : FAB+
RT : 12.69 min Scan# : (372,395)
Elements : C 69/0, H 71/0, O 18/0, In 1/0
Mass Tolerance : 1000ppm, 10mmu if m/z > 10
Unsaturation (U.S.) : 0.0 - 50.0

Observed m/z	Int%
1301.3638	100.00

	Estimated m/z	Err[ppm / mmu]	U.S.	C	H	O	In
1	1301.3601	+2.9 / +3.7	35.5	69	70	18	1

Fig S25. Mass Spectrum of DiMeOC-In.

Instituto de Química, UNAM
Laboratorios de Servicios Analíticos

Laboratorio de Análisis Elemental por Combustión

INFORME DE ANÁLISIS

Investigador solicitante: Dr. Raúl G. Enríquez Habib
Estudiante: William Meza Morales

No. de registro	Clave de la muestra	Valor	N [%]	C [%]	H [%]	S [%]	Fecha de análisis
155	DiMeOC-In	Teórico	---	63.70	5.35	---	23-06-2022
		Exp	---	63.33	5.23	---	
156	DiMeOC-Ga	Teórico	---	64.20	5.39	---	23-06-2022
		Exp	---	65.17	5.39	---	

Equipos:

Analizador elemental, marca Thermo Scientific, modelo Flash 2000.
Temperatura del horno: 950 ºC.
Microbalanza, marca Mettler Toledo, modelo XP6, calibrada y verificada.

Fig S26. Elemental analysis of complexes **2** and **3**.

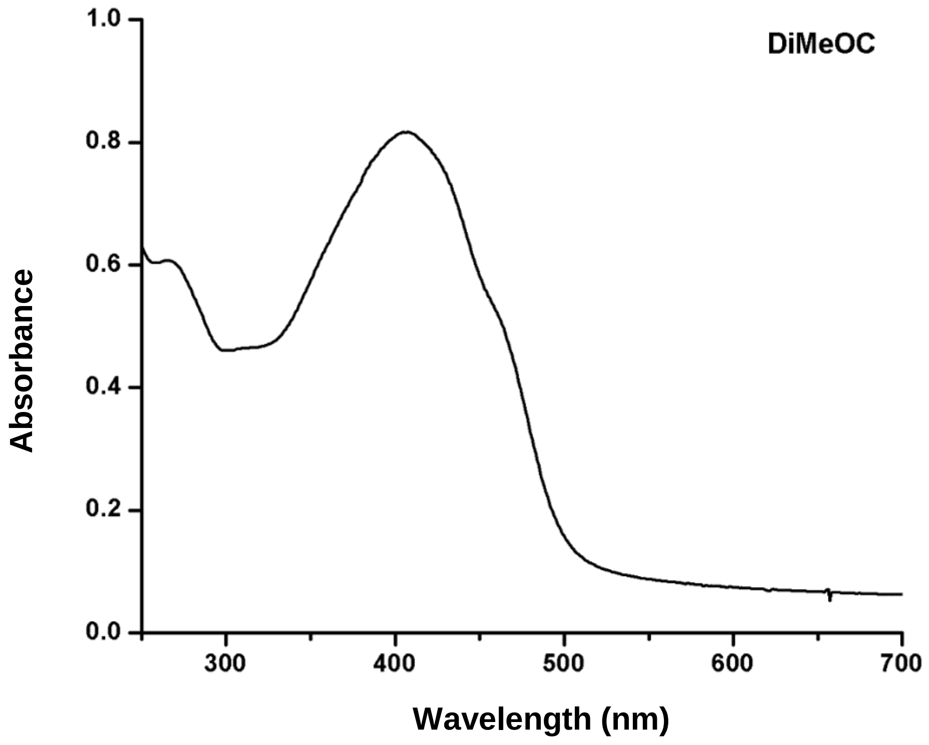

Fig S27. UV-Vis of DiMeOC.

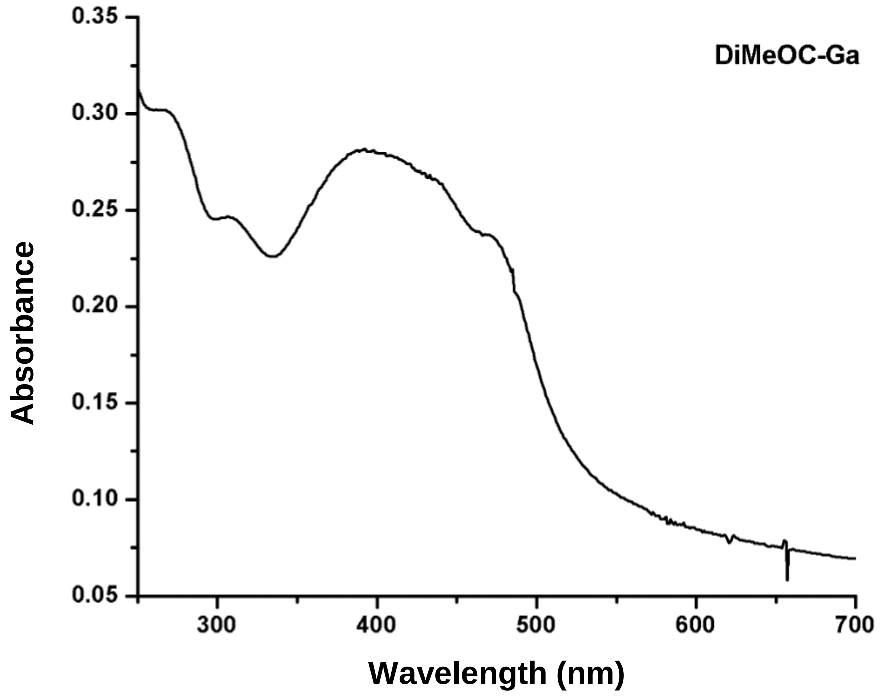

Fig S28. UV-Vis of DiMeOC-Ga.

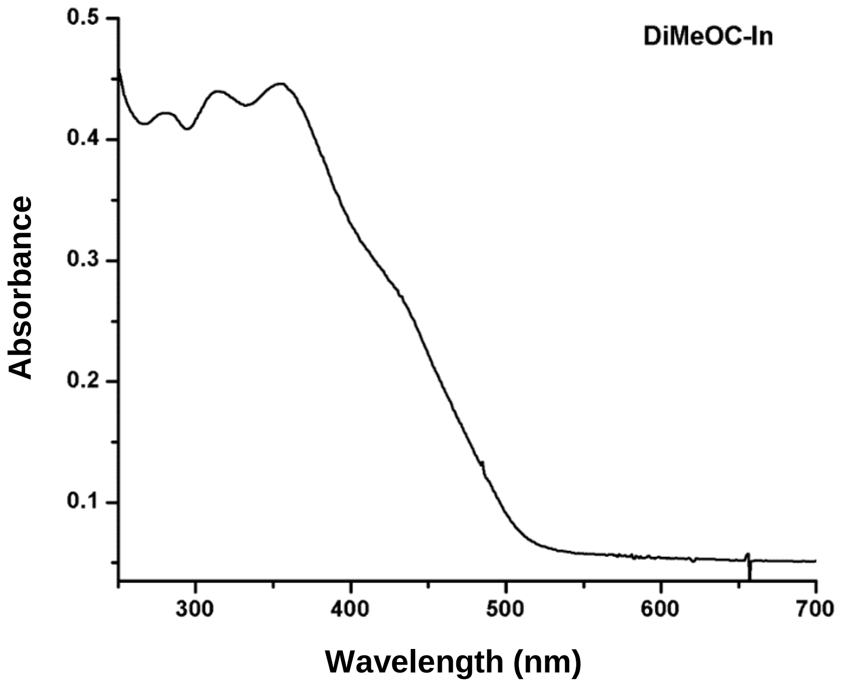

Fig S29. UV-Vis of DiMeOC-In.

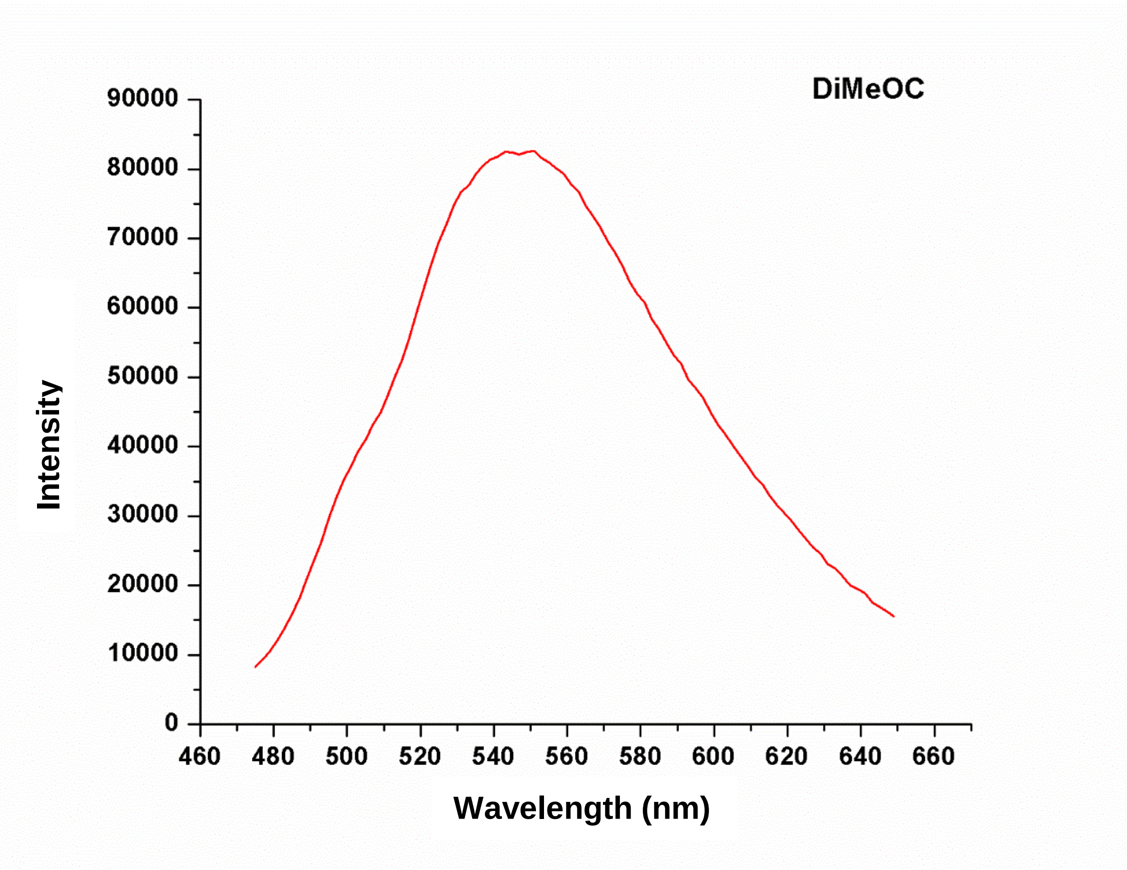

Fig S30. Emission spectrum of DiMeOC.

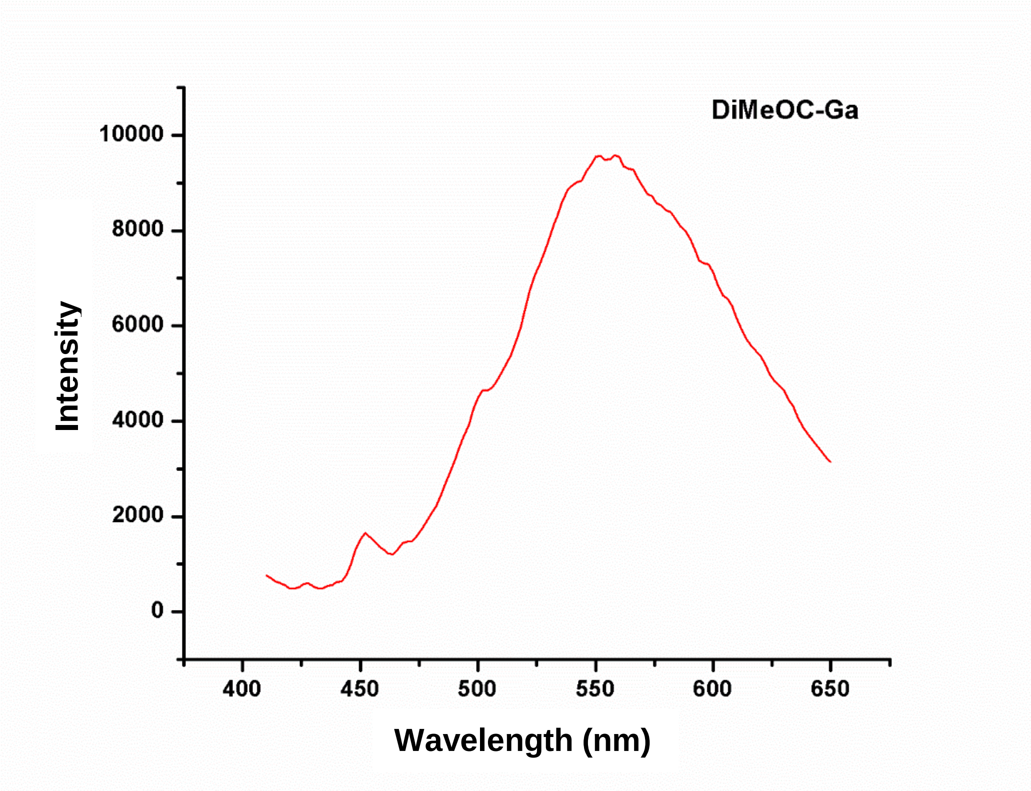

Fig S31. Emission spectrum of DiMeOC-Ga.

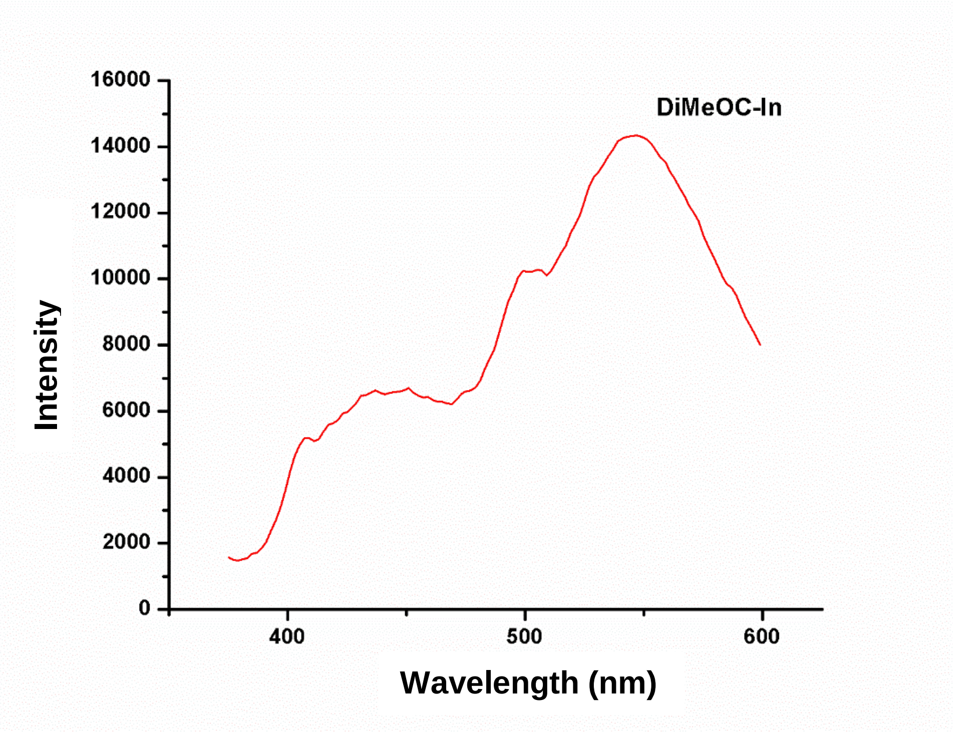

Fig S32. Emission spectrum of DiMeOC-In.

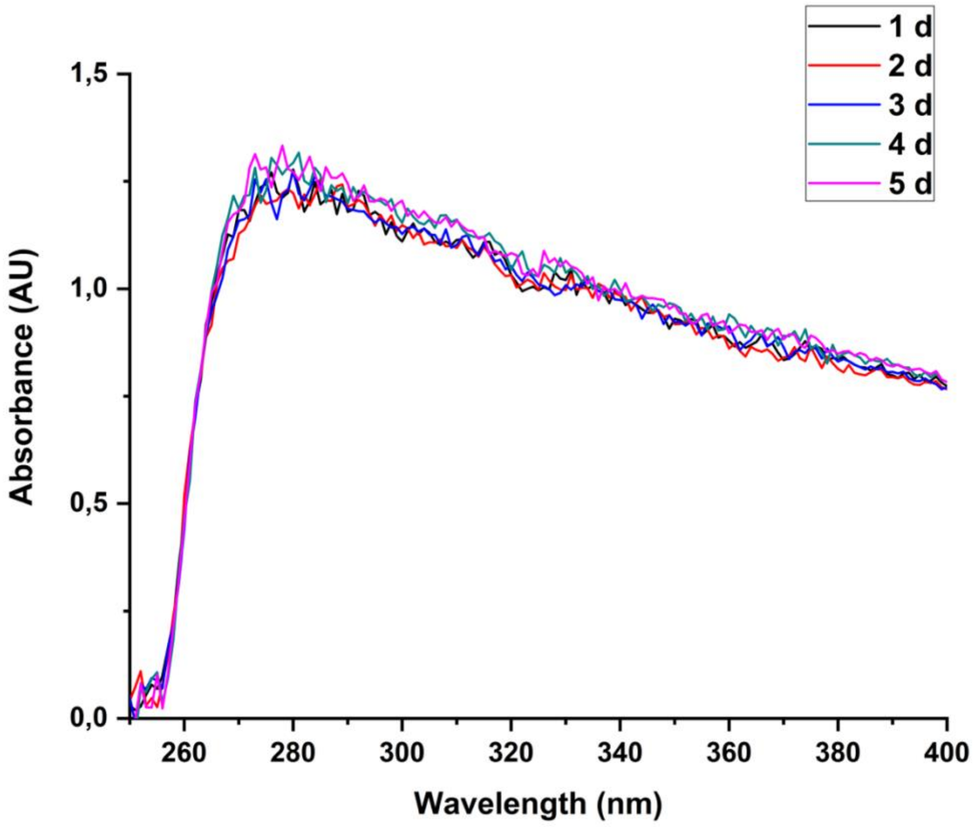

Fig S33. UV-Vis DiMeOC in water and 1% of DMSO (For 5 days).

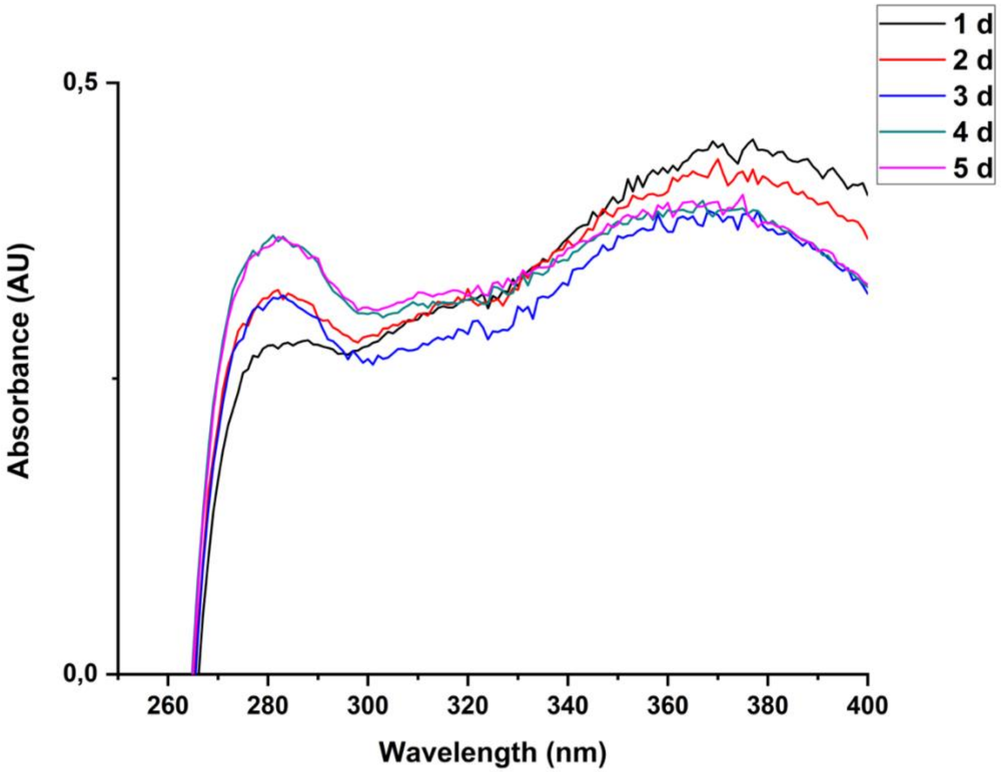

Fig S34. UV-Vis DiMeOC-Ga in water and 1% of DMSO (For 5 days).

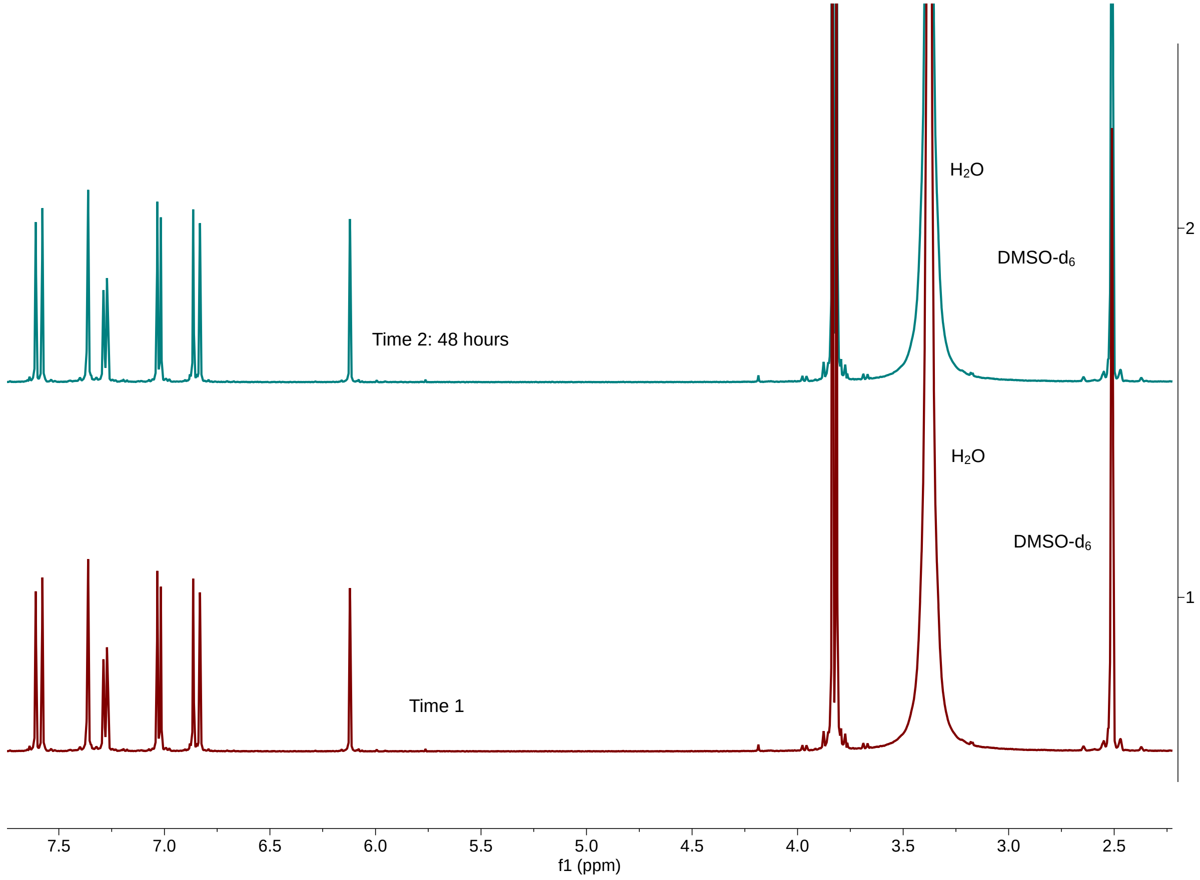

Fig S35. ^{1}H NMR DiMeOC in solution (DMSO-d_6, for 48 hours).

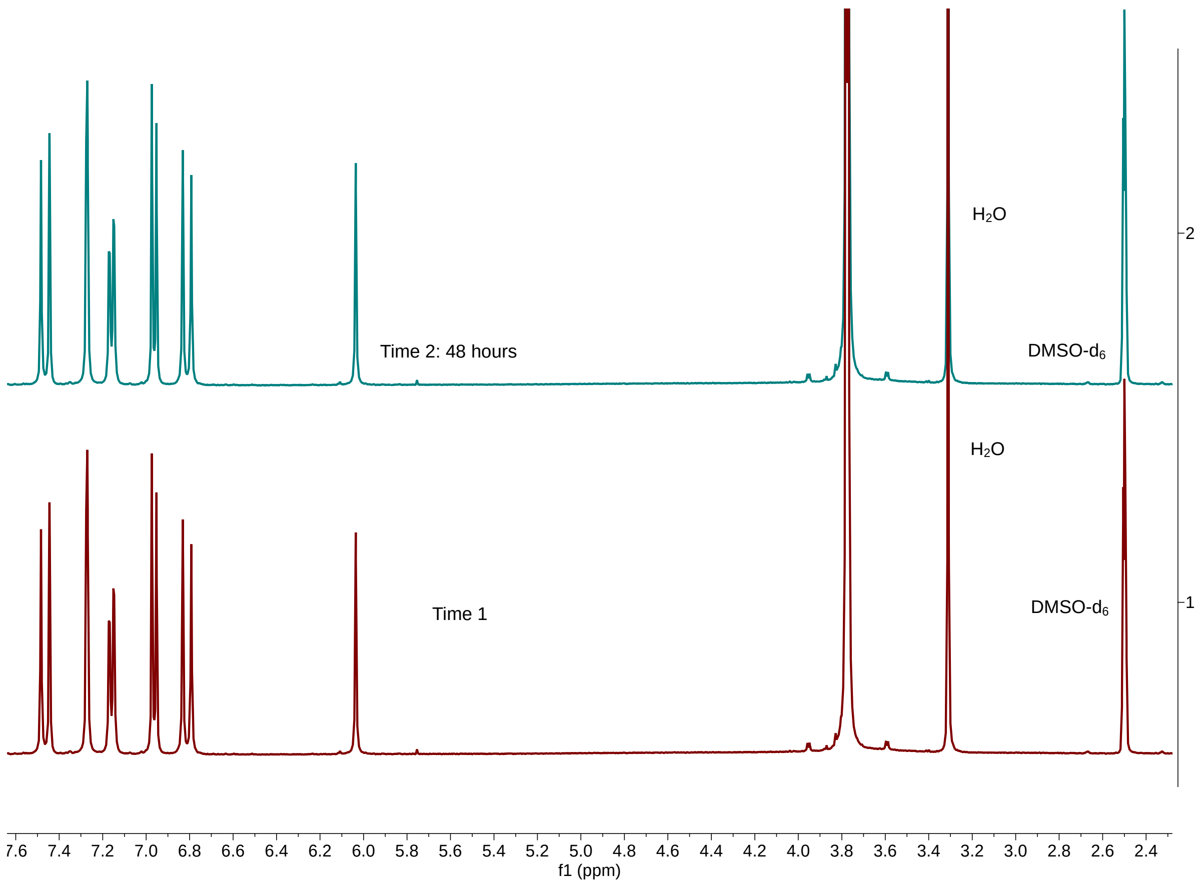

Fig S36. ^{1}H NMR DiMeOC-Ga in solution (DMSO-d_6, for 48 hours).

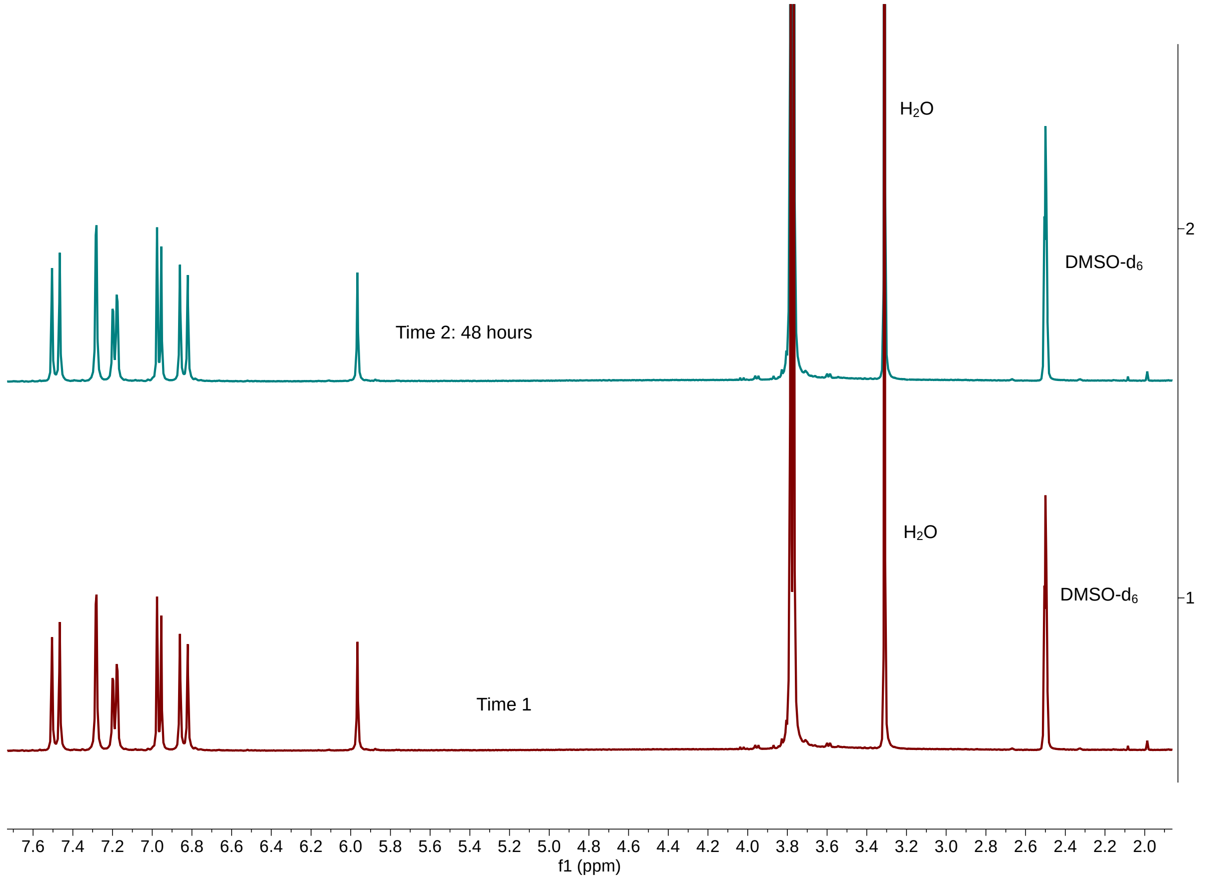

Fig S37. ^{1}H NMR DiMeOC-In in solution (DMSO-d_6, for 48 hours).
